# Multi-Level Computational Modeling of Anti-Cancer Dendritic Cell Vaccination Utilized to Select Molecular Targets for Therapy Optimization

**DOI:** 10.3389/fcell.2021.746359

**Published:** 2022-02-02

**Authors:** Xin Lai, Christine Keller, Guido Santos, Niels Schaft, Jan Dörrie, Julio Vera

**Affiliations:** ^1^ Laboratory of Systems Tumor Immunology, Department of Dermatology, Friedrich-Alexander-Universität Erlangen-Nürnberg (FAU) and Universitätsklinikum Erlangen, Erlangen, Germany; ^2^ Deutsches Zentrum Immuntherapie and Comprehensive Cancer Center Erlangen-EMN, Erlangen, Germany; ^3^ Departament of Biochemistry, Microbiology, Cell Biology and Genetics, Faculty of Sciences, University of La Laguna, San Cristóbal de La Laguna, Spain; ^4^ RNA Group, Department of Dermatology, Friedrich-Alexander-Universität Erlangen-Nürnberg (FAU) and Universitätsklinikum Erlangen, Erlangen, Germany

**Keywords:** melanoma, immunotherapy, gene networks, kinetic modeling, cellular therapy, bio-distribution, dendritic cell vaccine, systems medicine

## Abstract

Dendritic cells (DCs) can be used for therapeutic vaccination against cancer. The success of this therapy depends on efficient tumor-antigen presentation to cytotoxic T lymphocytes (CTLs) and the induction of durable CTL responses by the DCs. Therefore, simulation of such a biological system by computational modeling is appealing because it can improve our understanding of the molecular mechanisms underlying CTL induction by DCs and help identify new strategies to improve therapeutic DC vaccination for cancer. Here, we developed a multi-level model accounting for the life cycle of DCs during anti-cancer immunotherapy. Specifically, the model is composed of three parts representing different stages of DC immunotherapy – the spreading and bio-distribution of intravenously injected DCs in human organs, the biochemical reactions regulating the DCs’ maturation and activation, and DC-mediated activation of CTLs. We calibrated the model using quantitative experimental data that account for the activation of key molecular circuits within DCs, the bio-distribution of DCs in the body, and the interaction between DCs and T cells. We showed how such a data-driven model can be exploited in combination with sensitivity analysis and model simulations to identify targets for enhancing anti-cancer DC vaccination. Since other previous works show how modeling improves therapy schedules and DC dosage, we here focused on the molecular optimization of the therapy. In line with this, we simulated the effect in DC vaccination of the concerted modulation of combined intracellular regulatory processes and proposed several possibilities that can enhance DC-mediated immunogenicity. Taken together, we present a comprehensive time-resolved multi-level model for studying DC vaccination in melanoma. Although the model is not intended for personalized patient therapy, it could be used as a tool for identifying molecular targets for optimizing DC-based therapy for cancer, which ultimately should be tested in *in vitro* and *in vivo* experiments.

## Introduction

Dendritic cells (DCs) are the strongest stimulators of our immune response (Fong and Engleman, 2000). They are the most prevalent antigen-presenting cells in the immune system and regulate the systemic antigen presentation process. The ability to culture DCs *in vitro* and load them with exogenous antigens and their ability to subsequently activate cytotoxic T cell immunity makes them interesting candidates for cancer immunotherapy vaccines (Steinman, 1989; Timmerman and Levy, 1999; Bullock et al., 2003; Michiels et al., 2005; Morandi et al., 2006; Palucka and Banchereau, 2013; Schaft et al., 2013; Sprooten et al., 2019). Currently, two approaches to endow DCs with the antigenic T-cell epitopes are mainly pursued: exogenous peptides can be loaded directly onto the surface of the DCs, where they replace the endogenous peptides within the HLA-molecules. Alternatively, the antigens can be expressed within the DCs by mRNA transfection, to employ the natural antigen-processing machinery of the DCs, which generates epitopes from the encoded antigens and presents them in its HLA molecules (Sprooten et al., 2019). For instance, DCs are pulsed with tumor antigens in form of proteins or peptides (Timmerman and Levy, 1999) or electroporated or transfected with mRNA encoding tumor antigens to generate cancer vaccines (Michiels et al., 2005; Schaft et al., 2013). Specifically, the data showed that successfully transfected DCs express 10 times more antigens than those electroporated with tumor mRNAs and thus can activate more T cells (Schaft et al., 2013). Administered as a vaccine, DCs can induce protective anti-tumor immunity (Timmerman and Levy, 1999). Furthermore, some studies showed that after priming T cells with DCs transfected with tumor mRNA, T cells with both effector and memory phenotypes can be found and both the primary and the recall T-cell response are triggered (Bullock et al., 2003; Morandi et al., 2006).

Compared to other therapy approaches such as adoptive T-cell transfer, the DC therapy shows better tolerance in cancer patients to enhance immune response (Abbas et al., 2018). For example, it has been shown that adoptive transfer with tumor-reactive T cells in melanoma patients can result in tumor regression, but also induce an autoimmune response to normal tissues that led to inflammatory skin lesions (Yee et al., 2000; Dudley et al., 2002). In contrast, Schreurs et al. demonstrated that peptide-loaded DC vaccine can induce strong anti-tumor immunity and reduce toxicity of the immune therapy (Schreurs et al., 2000).

DCs can be derived *in vitro* from blood monocytes, loaded with tumor antigens and matured by cytokine-cocktails including TNFα, IL-1β, and IL-6 combined with PGE2 (Pfeiffer et al., 2014) to be subsequently injected into the patient in the form of a vaccine (Timmerman and Levy 1999; Fong and Engleman, 2000; Abbas et al., 2018). Immature DCs are triggered to mature by stimulation with TNFα or lipopolysaccharide (LPS). Upon maturation, the DCs become motile and travel from the tissue to the T-cell areas of peripheral lymphatic organs for the antigen presentation. They start secreting a variety of cytokines and chemokines (such as IL-6, IL-8, and IL-12) that serve as co-stimulators and attractants for the activation of CD8^+^ cytotoxic T cells. They also express surface molecules (e.g., CD70) that are used for the specialized interaction with the CD8^+^ T cells. CD8^+^ cytotoxic T lymphocytes are important effectors of anti-tumor immunity (Vesely et al., 2011; Abbas et al., 2018), and after the antigen presentation by DCs, successful stimulation of CD8^+^ T cells depends on the composition of these co-stimulatory factors such as cytokines and chemokines. The NF-κB signaling pathway is crucial for DC maturation (Tas et al., 2005; Morandi et al., 2006; Hernandez et al., 2007; Pfeiffer et al., 2014), and strategies targeting the pathway are continuously being developed to further improve this immunotherapy approach. One promising method, for example, is the electroporation of DCs with mRNA encoding constitutively active IKKβ that can activate the NF-κB signaling pathway and upregulate maturation markers such as CD40, CD70, CD80, OX40L, IL-12, and IL-8 leading to the persistent proliferation of CD8^+^ T cells with a memory phenotype (Pfeiffer et al., 2014).

In addition to experimental studies, researchers have developed computational models to study dynamic systems accounting for immunity against cancer. Such models not only help to dissect the molecular mechanism underlying immune response against cancer but also to design experiments to improve available anti-cancer immunotherapies. For instance, the model developed by Castiglione et al. describes the dynamics between DCs, CD8^+^ T cells, and tumor cells using a system of ordinary differential equations (ODEs). The model was used to search for an optimal protocol for the drug treatment, i.e., the optimal amount of DCs per vaccine, the optimal timing for one injection, and the optimal number of injections (Castiglione and Piccoli, 2007). Another work focuses on a personalized application using patient-specific parameters, and therefore, the interaction between immune effector cells and tumor cells was considered in the model (Kogan et al., 2012). Gong et al. used multiscale agent-based modeling to describe the dynamics between cytotoxic T cells and cancer cells and their three-dimensional distribution. The model provides a framework that enables predictions of treatment/biomarker combinations for different cancer types based on patient data (Gong et al., 2017). Mathematical modeling of T cell-macrophage interactions within the tumor microenvironment showed that inhibition of macrophage is the most effective strategy to promote T cell function, and therefore improving the effectiveness of immunotherapies that target macrophages (Cess and Finley, 2020). Arulraj and Barik developed an ODE model to investigate the role of feedback loops in inhibition of T-cell function by PD-1 and identified that the tyrosine kinase Lck is a crucial regulator for PD-1 induced inhibition of T-cell receptor signaling (Arulraj and Barik, 2018). De Pillis and coworkers developed a mathematical model describing the DC vaccination for melanoma and utilized it to propose therapy schedule that can improve the efficacy of the vaccine (DePillis et al., 2013). Santos and coworkers integrated transcriptomic data with mechanistic modeling of DC vaccination for melanoma to detect mechanisms that are related to sensitivity and resistance of the immunotherapy (Santos et al., 2016). So far, most of the published models have considered only cell-to-cell communications through direct contact or the secretion of cytokines and chemokines. However, intracellular biochemical networks that are crucial for regulating cell function can be tuned to improve immunotherapies (Lai et al., 2021). Therefore, integrating them into multi-level computational models offers the opportunity to simulate and analyze molecular events that can determine the efficiency of anti-cancer immunotherapies.

In this work, we developed a multi-level model accounting for DC-based anti-cancer immunotherapy. By calibrating, simulating, and analyzing the model, we aimed to understand the molecular mechanism and cell-to-cell interactions that are crucial for regulating DC-mediated immunogenic function and therefore identifying molecules that can improve the efficiency of the DC-based immunotherapy. Specifically, the model is composed of three parts representing different stages of the DC immunotherapy – the bio-distribution of the DCs in the human organs, the biochemical reactions regulating the DCs’ maturation, and DC-mediated activation of CD8^+^ T cells. Next, we calibrated the model using several experimental data sets accounting for the dynamics and bio-distribution of intravenously injected DCs in the human body, the kinetics of molecules during DC maturation, and the dynamics of the T cell population after the injection of the DC vaccine, respectively. Then, we performed sensitivity analysis on model parameters to identify molecules and biochemical reactions that are impactful on a DC-mediated T-cell response. We found the NF-κB regulators (i.e., IKKβ and IκBα) and cytokines (i.e., IL-6 and IL-8) are top-ranking molecules for regulating the T-cell response. Finally, we ran simulations to quantify how modulating the expression of the identified molecules can change the number of memory T cells. Such results lay the basis for experimental validation of the effects of the identified molecules for improving the efficiency of DC immunotherapy. Taken together, the modeling approach allows for the effective integration of experimental data into a multi-level model accounting for DC-based anti-cancer immunotherapy. Although the model is not intended for personalized patient therapy, it could be used as a tool for identifying molecular targets for optimizing DC-based therapy for cancer, which ultimately should be tested in *in vitro* and *in vivo* experiments.

## Materials and Methods

### Model Construction and Simulation

We developed a multi-level model accounting for three stages of DC immunotherapy – the spreading and distribution of intravenously injected DCs in the human organs, the biochemical reactions regulating the DCs’ maturation and activation, and DC-mediated T-cell responses. The model was implemented using ODEs and simulated in MATLAB R2015b (*see* Supplementary Material for details). We used the MATLAB function *ode45* to solve the system accounting for the maturation of DCs. The function is based on the Dormand and Prince Runge-Kutta methods. We used the MATLAB function *dde23* to solve the equations accounting for a DC-mediated T-cell response. This method is based on an explicit Runge-Kutta method pair and especially for delay differential equations with constant delay. We ran simulations on a computer with a four core CPU (3.2 GHz) and 8 GB RAM.

Here, we listed some representative equations for each part of the model. Specifically, we used four ODEs to describe the temporal dynamics of DCs in the blood, the spleen, the liver, and the lung. A representative equation is shown as follows

$$\frac{d}{dt}DC_{Spleen}\left(t\right)=\mu_{BS}\frac{Q_{Blood}}{Q_{Spleen}}DC_{Blood}\left(t\right)-\mu_{S0}DC_{Spleen}\left(t\right)$$ (1)

The number of DCs in the spleen ( $DC_{Spleen}$ ) is determined by the flow of DCs from the blood ( $DC_{Blood}$ ) into the spleen at the rate ( $\mu_{BS}$ ) and its degradation rate ( $\mu_{S0}$ ). $Q_{Blood}$ and $Q_{Spleen}$ denote the volume of the blood and spleen, respectively.

The maturation of DCs in the spleen is characterized by 20 ODEs that account for the activation of the NF-κB pathway and its downstream targets such as cytokines that are crucial for T-cell responses. The equations accounting for the mRNA and protein of IL-8 are shown below

$$\frac{d}{dt}mIL8\left(t\right)=k_{transc1}^{mIL8}+k_{trancs2}^{mIL8}\cdot NF\kappa B\left(t\right)-k_{deg}^{mIL8}\cdot mIL8\left(t\right)$$ (2)

$$\frac{d}{dt}IL8_{DC}\left(t\right)=k_{transl}^{IL8}\cdot mIL8\left(t\right)-k_{deg}^{IL8}\cdot IL8_{DC}\left(t\right)-k_{sec}^{IL8}\cdot IL8_{DC}\left(t\right)$$ (3)

The transcription of IL-8 mRNA is determined by its basal transcription rate ( $k_{transc1}^{mIL8}$ ) and another term ( $k_{trancs2}^{mIL8}\cdot NF\kappa B\left(t\right)$ ) accounting for the regulation by NF-κB. $k_{deg}^{mIL8}$ denotes the degradation of the IL-8 mRNA. The protein expression of IL-8 is determined by its translation from its mRNA ( $k_{transl}^{IL8}$ ), its degradation ( $k_{deg}^{IL8}$ ), and its secretion ( $k_{sec}^{IL8}$ ) from DCs into the spleen.

The activation of T cells is modeled using four ODEs. The equations describe the process of how lymph node T cell activation translates to different phenotypes of T cell subsets such as early effector, short-lived effector, and memory T cells. The equation describing memory T cells is shown as follows

$$\frac{d}{dt}M\left(t\right)=k_{diff2}^{EE}\cdot EE\left(t\right)+0.1\cdot k_{deg}^{SLE}\cdot SLE\left(t\right)$$ (4)

The number of memory T cells (*M*) is translated from early effector (*EE*) and short-lived effector (*SLE*) T cells with the rates of $k_{diff2}^{EE}$ and $k_{deg}^{SLE}$ , respectively. The constant 0.1 denotes that in experiments only 10% of the short-lived effector T cells become memory T cells (Badovinac et al., 2002; Mueller et al., 2013).

### Structural Identifiability Analysis

Before model calibration, it is useful to investigate whether it is possible to obtain identifiable parameters using experimental data. Global structural identifiability analysis can provide a good indication of this. Therefore, we used the MATLAB toolbox GenSSI to perform structural identifiability analysis (Chis et al., 2011). The algorithm uses Lie derivatives of the ODE model to investigate the structural identifiability of ODE models. Theoretically, if sub-models are structurally identifiable, so is the entire model (Villaverde et al., 2016). Therefore, to reduce computation time we divided our model into separated parts, including DC distribution, NF-κB activation, NF-κB-mediated secretion of cytokines, and T-cell response, and performed the analysis on each part.

### Model Calibration

We used a hybrid method that combines global and local optimization algorithms to perform parameter optimization. Such a method facilitates global exploration of parameter space and fast local convergence (Villaverde et al., 2019). Specifically, we derived 1000 parameter sets using Latin hypercube sampling that not only samples random parameter values but also guarantees a uniformed distribution of parameter values in their defined ranges (Tang, 1993). The 1000 parameter sets are initial values of model parameters and were used for model calibration. We first fit the model to the experimental data using the pattern search algorithm (MATLAB function *patternsearch*) that is a global derivative-free optimization algorithm. The top 100 the solutions of the global optimization results (quantified by the cost function) were used for subsequent local optimization. The local optimization algorithm (MATLAB function *fmincon*) is gradient-based and allows for efficient searching that makes the cost function converge fast. We obtained the optimum parameter set that minimizes the cost function

$$\Phi \left(p\right)=\underset{i,j}{\sum }{\left(\frac{y_{i}\left(t_{j}\right)-y_{i}\left(t_{j},p\right)}{\underset{j}{max}\left(y_{i}\left(t_{j}\right)\right)\cdot sd\left(y_{i}\left(t_{j}\right)\right)}\right)}^{2},$$ (5)

Where $y_{i}\left(t_{j}\right)$ represents the experimental data that shows the value of the observation $y_{i}\left(t_{j}\right)$ at time point *j*. $y_{i}\left(t_{j},p\right)$ is the corresponding model simulation with a specific set of parameter values *p*. The cost function is normalized using the maximum value of each experimental data set (i.e., $\underset{j}{max}\left(y_{i}\left(t_{j}\right)\right)$ ) to prevent the biased effects caused by different data scaling during parameter estimation. If available, the cost function is additionally weighted by the standard deviation of the experimental data $sd\left(y_{i}\left(t_{j}\right)\right)$ .

We used the experimental data accounting for *in vitro* differentiated DCs into the liver, the spleen, the lung and other periphery after intravenous injection (Mackensen et al., 1999) to characterize the dynamics of DCs in human organs (Supplementary Table S1). We characterized NF-κB pathway activation in DCs (Supplementary Table S2), cytokine and chemokine production by DCs (Supplementary Table S2), and DC-mediated T cell responses (Supplementary Table S3) using the data from our previous publication (Pfeiffer et al., 2014). A detailed description of how each part of the model is calibrated can be found in Supplementary Material.

### Practical Identifiability Analysis and Confidence Intervals of Parameters

To analyze the uncertainty in parameter estimates, we computed Pearson correlation coefficients to quantify their linear dependence using the top 100 out of 1000 estimates. Among the top 100 parameter estimates, we used the best 15 parameter estimates that show the minimum value in the cost function to calculate the confidence intervals of estimated parameters. Specifically, for each estimated parameter we generated 1000 bootstrap samples using its estimated values in the best 15 parameter estimates. Then, we used the mean value and standard deviation of the 1000 samples to derive the parameters’ 95% confidence intervals. We performed the analyses using the MATLAB function *bootci*.

### Sensitivity Analysis

We performed global sensitivity analyses to quantify the impact of model parameters on the dynamics of the system. We used the Sobol method that considers variations within the entire variability space of the model parameters (Saltelli et al., 2008; Sarrazin et al., 2016). We computed two types of indices: first-order indices (main-effects) and total-order indices (total-effects). The former measures the direct contribution from a model parameter (e.g., the total amount of NF-κB) to a model variable (e.g., the count of memory T cells), while the latter measures the overall contribution including the direct contribution and the amplification of this contribution due to interactions with all other model parameters (Sarrazin et al., 2016). The analysis was performed using the MATLAB toolbox *SAFE* (Pianosi et al., 2016) and the detailed computation of the sensitivity indices can be found in Supplementary Material.

## Results

### The Multi-Scale Model Accounting for DC-Based Anti-cancer Immunotherapy

We developed a multi-scale model accounting for DC-based anti-cancer immunotherapy. We considered different stages of the DC therapy: *1*) the bio-distribution of DCs in the human body after the treatment, *2*) the biochemical pathways underlying DC maturation, and *3*) the DC-induced immune response such as activation of CD8^+^ T cells.

**FIGURE 1:**
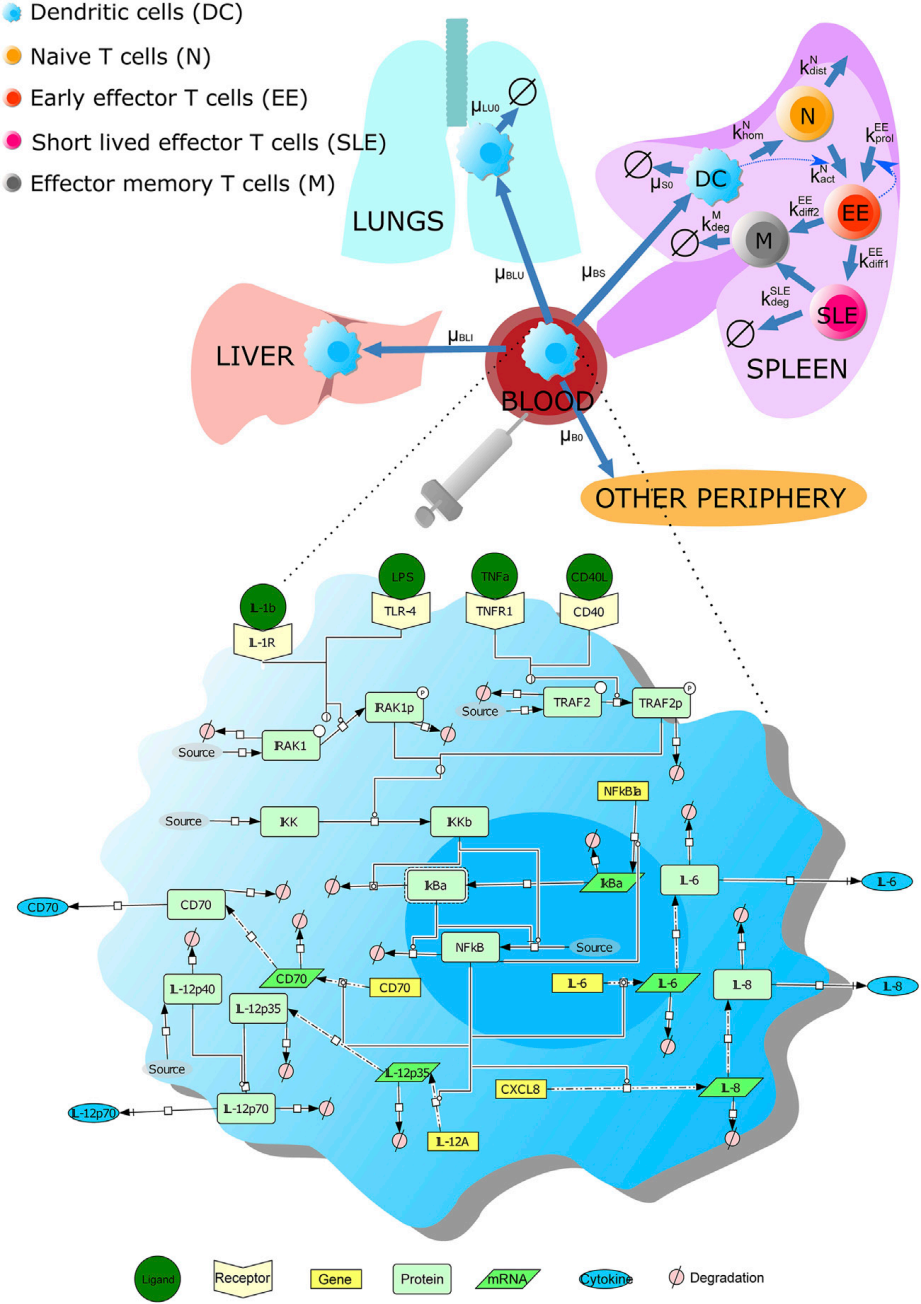
Scheme of the multi-scale model accounting for DC vaccine against cancer. The multi-level model contains three parts: the kinetics and bio-distribution of intravenously injected DCs in human organs (including the liver, the lung, and the spleen), the signaling pathways underlying DC maturation, and DC-mediated T-cell response. The labels next to the blue arrowed lines are the corresponding model parameters. The signaling pathway in DCs is drawn using Systems Biology Graphical Notation. A detailed description of the model can be found in Supplementary Material.

We characterized the trafficking and distribution of dendritic cells (DCs) in the human body using published data (Ludewig et al., 2004) (Figure 1; *see* Supplementary Material for details). Specifically, DCs are administrated into patients through intravenous injection. The injected DCs mainly spread into the liver, the lung, the spleen, and other peripheries. According to the data (Mackensen et al., 1999; Ludewig et al., 2004), after reaching the liver, DCs reside there, while DCs enter quickly into the lung but also decrease to a minimal level. In our model, we used the spleen as a representative lymphoid organ, in which effective, antigen presentation-mediated interactions between T and dendritic cells happen (Mackensen et al., 1999; Barinov et al., 2017; Abbas et al., 2018).

Concerning the pathways underlying DC maturation and activation, we further considered in the model the NF-κB pathway underlying DC maturation; this part of the model was adapted from our previous results (Schulz et al., 2017) (Figure 1; *see* Supplementary Material for details). In our model, mature DCs secrete a variety of cytokines (e.g., IL-12, IL-6, and IL-8) that can lead to T cell activation. The production of the cytokines is due to the stimulation of receptors (such as TNFα receptor, IL-1 receptor, CD40 receptor, and TLR4), leading to the activation of the NF-κB signaling pathway. Besides, we considered a surface protein CD70 expressed by DCs after stimulation, as the protein can induce the expansion of antigen-specific CD8^+^ T cells.

Finally, we included a model module accounting for T cell activation by DCs in the spleen. The process was modeled considering three phases: *1*) a short-term interaction between the naive T cells and DCs; *2*) upregulation of activation markers and initiation of IFNγ and IL-2; and *3*) T cell proliferation after contact with DCs.

Taken together, we developed a model accounting for the life cycle of intravenously injected DCs from their spreading in human organs to inducing a T-cell response in the spleen. The resulting model contains 25 variables and 46 parameters (*see* Supplementary Material for details). The model includes not only cell population dynamics in human organs but also biochemical reactions that are crucial for DC maturation, making it an *in silico* platform to investigate intracellular manipulation of DCs utilized in vaccination against the tumor.

### Model Calibration Using Experimental Data

After constructing the multi-scale model, we performed structural identifiability analysis to identify whether model parametrizations with different values can produce the same simulations results (*see* Materials and Methods). The results showed that the whole model is not structurally identifiable. Specifically, the DC distribution part of the model is structurally non-identifiable, and this is caused by the parameters accounting for volumes of human organs (*Q*_*Blood*_, *Q*_*Spleen*_, *Q*_*Lung*_, and *Q*_*Liver*_), So, we fixed their values using the physiological information (Supplementary Table S1). The part accounting for NF-κB activation is structurally non-identifiable, and this is caused by parameters accounting for the phosphorylation rate of IRAK1 ( $k_{ph2}^{IRAK1}$ ) and TRAF2 ( $k_{ph2}^{TRAF2}$ ). Hence, we made their values equal to other phosphorylation processes catalyzed by other enzymes (Supplementary Table S2). The other model parts are structurally identifiable, and they account for NF-κB-mediated secretion of cytokines and chemokines and T cell response.

Next, we characterized the model parameters using published experimental data sets (*see* Materials and Methods). We separately calibrated the model using independent datasets that account for the dynamics of the system at different levels. Such a strategy is suitable and has been used for biological models composed of parts with different structural, time, and space scales (Vera et al., 2013).

**FIGURE 2:**
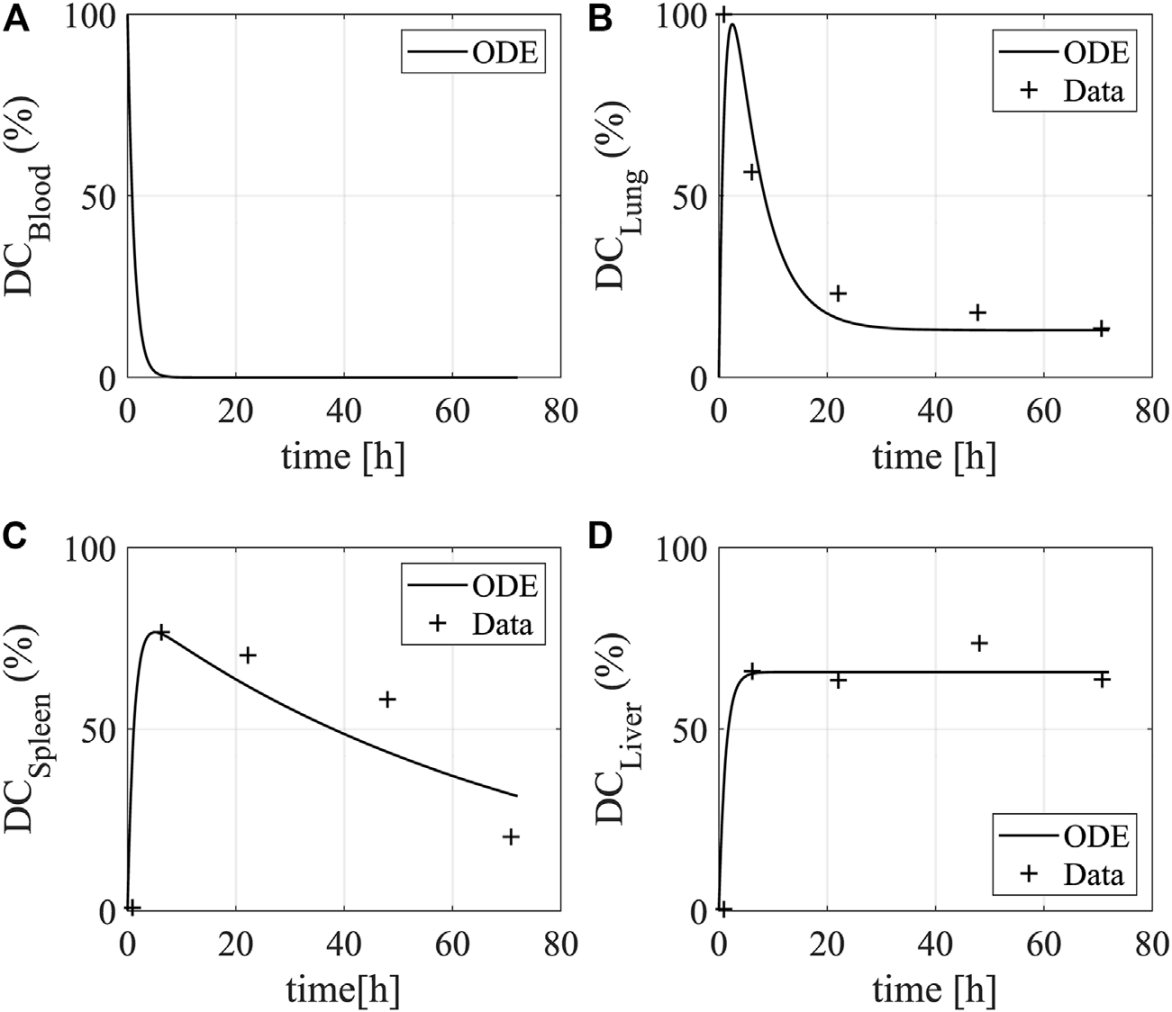
Dynamics of DCs in human organs. The plots show the bio-distribution and kinetics of DCs in **(A)** the blood **(B)** the lung, **(C)** the spleen, and **(D)** the liver. The data show the uptake (y-axis) of intravenously injected DCs over time (x-axis). The lines and star asterisks denote model simulations and experimental data, respectively. The experimental data is from Figure 2 in Mackensen et al. (1999). The temporal distribution of DCs were quantified by radioactivity in the lung, spleen, and liver of a patient after intravenous injection for 72 h.

We used the data that quantify injected DCs’ activities in the lung, liver, spleen, and blood to characterize model parameters associated with the trafficking and distribution of DCs in the human body (Mackensen et al., 1999). The obtained model can reproduce the data available (Figure 2). After DCs are injected into the blood, they quickly spread into the other organs and the amount of DCs decreases to zero in the blood. DCs traffic into the lung leading to a temporal increase followed by a quick drop, and afterward, the DCs stay at a low level. DCs enter the liver and remain stable in number for up to 72 h (Mackensen et al., 1999). In the spleen, the amount of DCs rapidly reaches a peak and gradually decreases as they circulate in the body after provoking a T-cell response.

**FIGURE 3:**
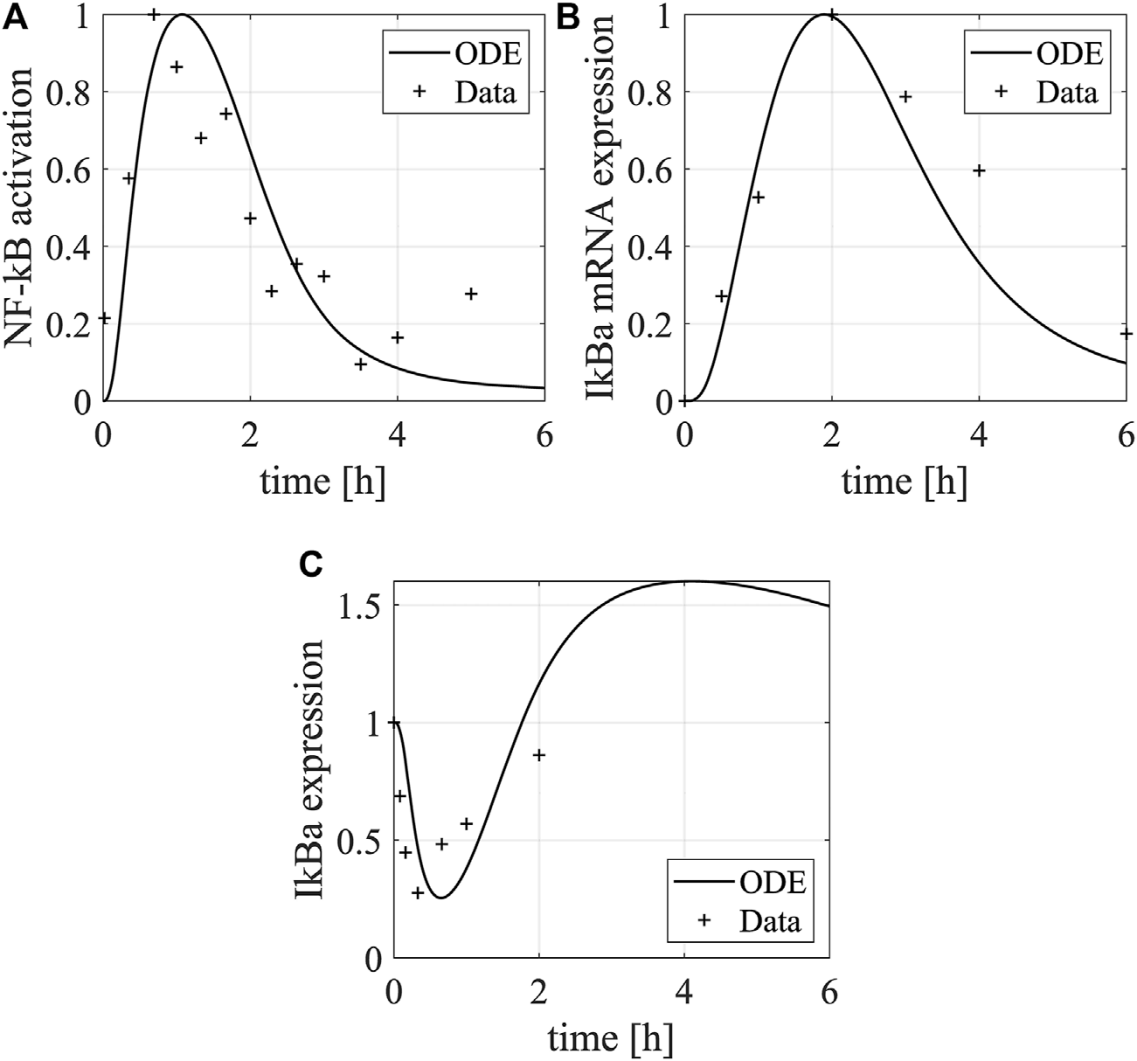
Dynamics of IκBα and NF-κB in DCs. The plots show the dynamics of **(A)** NF-κB protein and **(B)** IκBα mRNA and **(C)** protein in DCs after LPS stimulation. The lines and asterisks denote model simulations and experimental data, respectively. The NF-кB activation was characterized by its binding activity to DNA, and the experimental data were normalized to the maximal binding activity (Figure 3C in Bode et al., 2009). The data shown here is a representative of three independent experiments. The IκBα mRNA was measured by qPCR (Figure 5A in Bode et al., 2009) and its relative mean expression (normalized to the maximal value) in comparison to the mRNA encoding the house-keeping gene β-actin was shown. The IκBα protein expression was quantified using a representative western blot (Figure 3A in Bode et al., 2009) and normalized to the maximal value. The western blot data was quantified using the software ImageJ.

**FIGURE 4:**
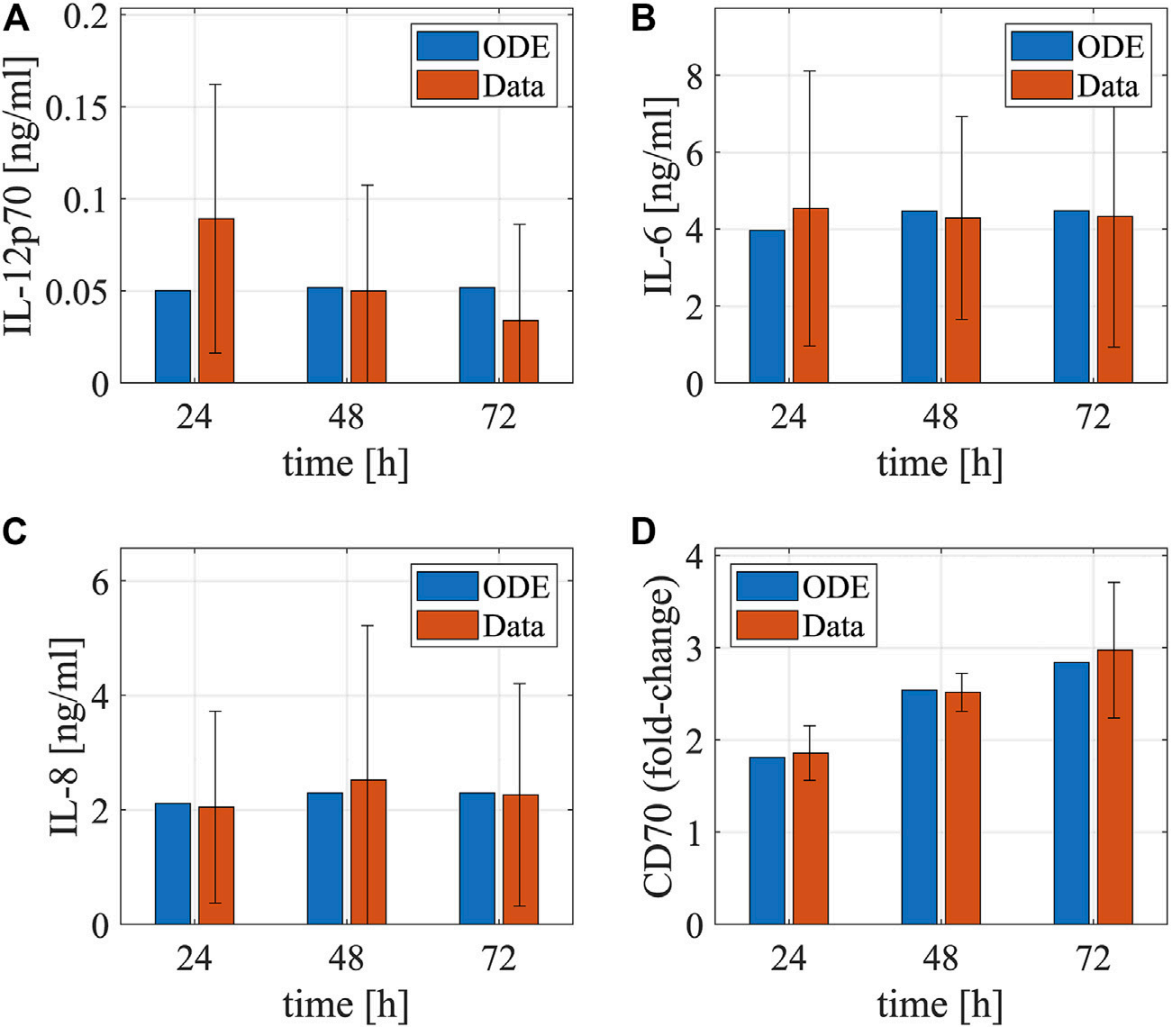
Dynamics of cytokines and membrane proteins in DCs. The bar plots show the temporal concentrations of **(A)** IL-12, **(B)** IL-6, **(C)** IL-8, and **(D)** CD70 after electroporating DCs with mRNAs encoding constitutively active IKKβ. The red and blue bars denote model simulations (the best fitting) and experimental data (mean ± standard deviation), respectively. The experiments were repeated for four times. The cytokine data are from Figure 3A in Pfeiffer et al. (Pfeiffer et al., 2014). The matured DCs’ cytokine concentrations after electroporation of IKKβ mRNA in the supernatants were determined by cytometric bead array. The data were measured using samples from eight different donors at the respective time points. The CD70 data is from Figure 1B in Pfeiffer et al. (Pfeiffer et al., 2014). It was assessed by flow cytometry in matured DCs electroporated with IKKβ mRNA. Fold-changes of CD70 compared to the controls (no electroporation of IKKβ mRNA) were calculated using the mean fluorescence intensity.

As the model equations accounting for the NF-κB pathway underlying DC maturation were adapted from our previous model, we used their original parameter values as initial values and re-calibrated them by fitting the model simulations to the experimental data measuring NF-κB pathway activation after LPS stimulation (Bode et al., 2009). Using the data, we characterized the dynamics of IκBα mRNA and protein and NF-κB in DCs (Figure 3). The LPS stimulation upregulates the expression of free NF-κB, as the increased IKKβ by the stimulation releases NF-κB from the complex formed by NF-κB and IκBα and degrades IκBα through phosphorylation. The free NF-κB promotes the transcription of the IκBα mRNA, leading to the recovery of IκBα that downregulates the expression of free NF-κB through a negative feedback loop. Besides, we used another set of data to characterize the dynamics of cytokines and surface markers after electroporating DCs with RNAs encoding constitutively active IKKα and -β (caIKK) that can activate the NF-κB pathway (Pfeiffer et al., 2014). Such treatment results in upregulation of IL-12, IL-6, IL-8, and CD70 for 72 h (Figure 4) and these markers are crucial for priming a T-cell response.

**FIGURE 5:**
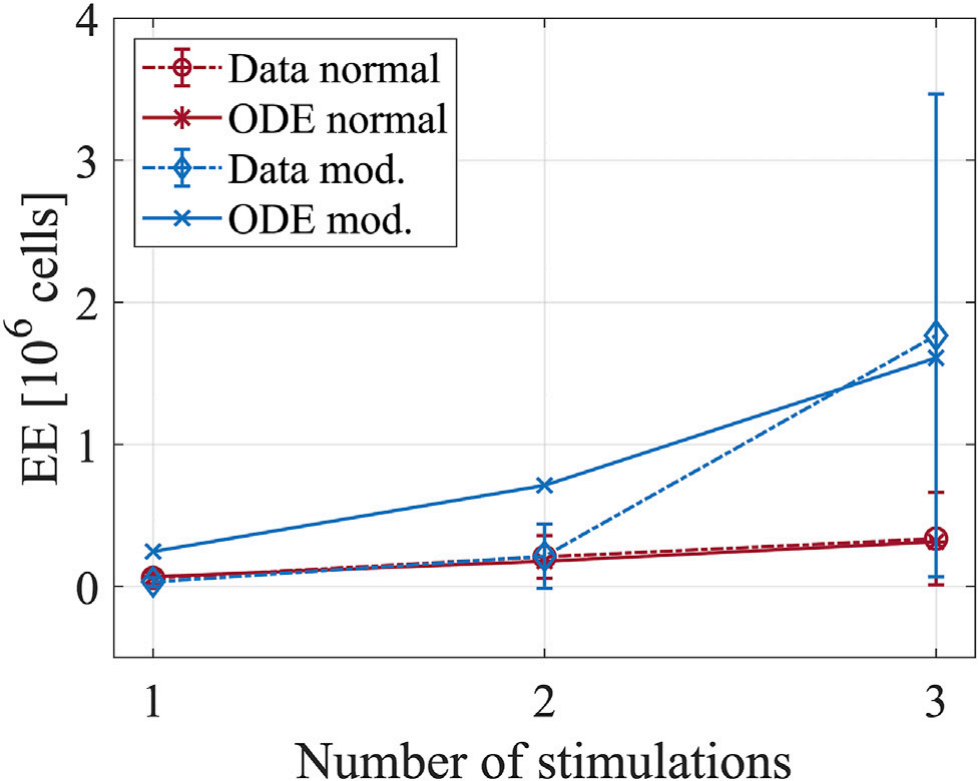
Dynamics of T-cell populations after co-culturing them with DCs. The plot shows the number of short-lived effector cells after priming with mock-electroporated DCs (red lines) and DCs electroporated with mRNAs encoding constitutively active IKKβ (blue lines). The experimental data is from Figure 4B in Pfeiffer et al. (Pfeiffer et al., 2014). Four hours after electroporation, the DCs were used to stimulate MelA-specific CD8^+^ T cells. In total, three stimulations were performed with an interval of 1 week between two subsequent stimulations. After each stimulation, the number of T cells was determined by tetramer-staining.

To characterize the model parameters that are associated with T-cell priming by DCs in the spleen, we used the *in vitro* data that show dynamics of the T-cell population after co-culturing them with control DCs or caIKK-DCs (Pfeiffer et al., 2014). Compared to the control DCs, the caIKK-DCs secret more cytokines such as IL-12, IL-8, IL-6, and TNF (Pfeiffer et al., 2014) and increase the production of T cells (Figure 5).

**FIGURE 6:**
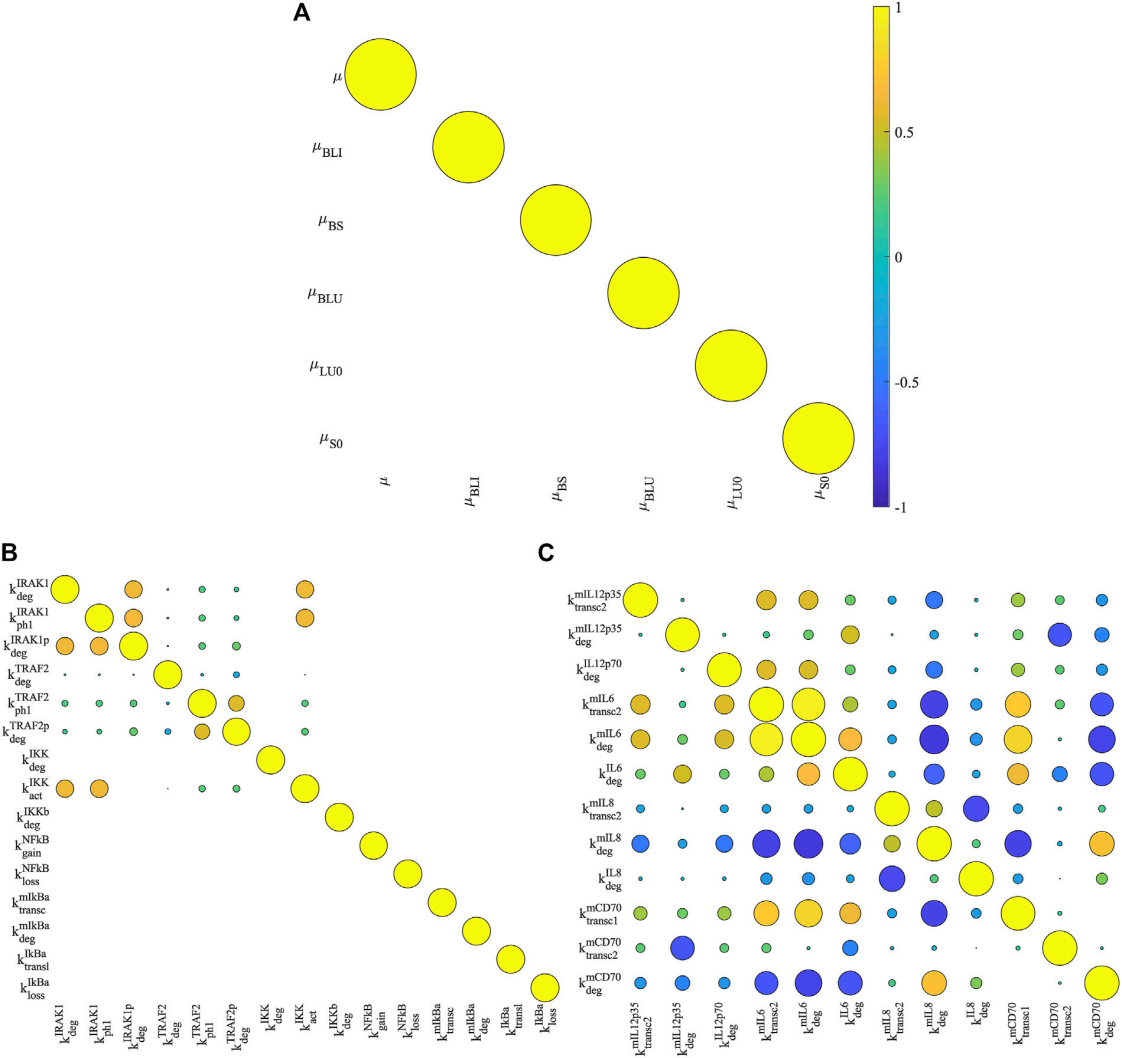
Correlation analysis of model parameters. We computed the Pearson correlation coefficients for estimated model parameters of bio-distribution of DCs **(A)**, signal transmission **(B)** and cytokine production **(C)** in NF-κB pathway. The correlation coefficients are visualized by circles. Their values are proportional to the size of the circles, and their signs are denoted by colors (positive: yellow; negative: blue). A value of zero (empty grids) means that two parameters are not correlated because either or both of their estimated values are unique.

Furthermore, we performed practical identifiability analysis in parameter estimates (*see* Materials and Methods). This allowed us to examine whether the estimated parameters are practically identifiable – the estimated model parameters have unique values that fit model simulations to experimental data used for model calibration. As shown in Figure 6A, the estimated parameters for DC distribution have no correlation with each other suggesting the corresponding parameters are practically identifiable. This is confirmed by the distribution of estimated parameter values in the best 15 parameter estimates that show the minimum cost function value (Supplementary Figure S1). All estimated parameters for DC distribution have unique values for the best parameter estimates. In addition, the model parameters accounting for the NF-κB pathway underlying DC maturation show moderate correlations (Figures 6B,C). Among the 28 estimated parameters, five are practically non-identifiable and they are $k_{ph1}^{TRAF2}$ , $k_{deg}^{TRAF2p}$ , $k_{deg}^{mIL6}$ , $k_{transc2}^{mIL8}$ , and $k_{deg}^{IL8}$ (Supplementary Figure S2). The non-identifiable parameters show small variances in their estimated values (Supplementary Table S2). This is due to the relatively small number of experimental data that are available for parameter estimation (Raue et al., 2009). The Michaelis-Menten coefficient *K*_*4*_ is the only parameter estimated to fit model simulations to the data accounting for T-cell dynamics (Supplementary Table S3). The estimated value of *K*_*4*_ is practically identifiable for its unique value in the best parameter estimates (Supplementary Figure S3). Taken together, most model parameters are practically identifiable because of their unique estimated values in the best parameter estimates. The practically non-identifiable parameters have confidence intervals with small ranges, suggesting minor influences on their biological interpretability.

Due to the lack of a complete data set that can characterize the dynamics of DCs used as an anti-cancer therapy, we calibrated model modules separately using relevant datasets from different publications. In all cases, the datasets were produced using human material or relevant experimental models that are generally accepted in the context of DC vaccine development (Brossart et al., 2001). We think they are complementary because they characterize the dynamics of the DC vaccination at different levels. This strategy has been used in other data-driven computational models (Sobotta et al., 2017, Hesse et al., 2021, Fey et al., 2015).

### Identification of Crucial Parameters Affecting DC-Mediated T-Cell Responses

After finishing calibrating the model with experimental data, we used it to simulate a DC-mediated T-cell response. Before running simulations, we set *DC*_*in*_ = 10^5^ as the data showed that about 10^5^ DCs are required for a T-cell response with a 70% probability (Celli et al., 2012). We set *Q*_*Spleen*_ = 10^5^ as the measured volume of a spleen is 10^5^ mm^3^ (Odorico et al., 1999). Besides, we assumed that the initial number of antigen-specific naive T cells in the spleen is 10^6^, so we set *T*_*0*_ = 10^6^ (Celli et al., 2012, Henrickson et al., 2008). We simulated the dynamics of different T cells in the spleen after injection with two different DC vaccines: normal DCs and DCs electroporated with caIKKβ-RNA (caIKK-DCs). In the simulations, both types of DCs were injected at *t* = 0 h with different degradation rates of IKKβ that are caused by caIKK in DCs ( $k_{deg}^{IKKb}$ = 0.840 h^−1^ for normal DCs and $k_{deg,mod}^{IKKb}$ = 0.216 h^−1^ for caIKK DCs).

**FIGURE 7:**
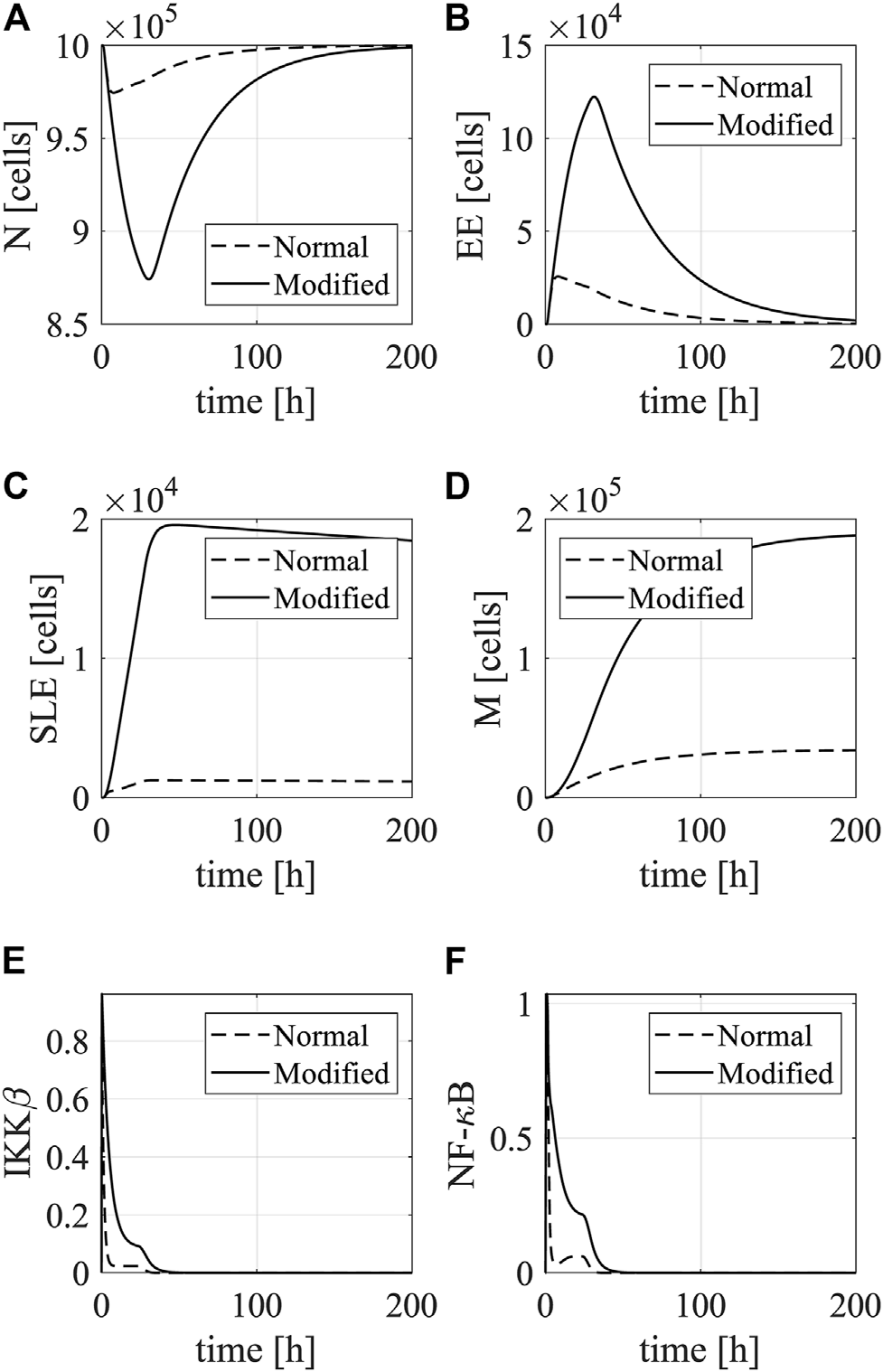
Simulations of DC-mediated T-cell response. The plots show dynamics of **(A)** Naive T cells, **(B)** early effector T cells, **(C)** short-lived effector T cells, and **(D)** memory T cells in the spleen after stimulation with mock-electroporated DCs (dotted line) and caIKKβ-mRNA-electroporated DCs (solid line). Besides, we show the dynamics of **(E)** IKKβ and **(F)** NF-κB in DCs (non-dimensionalized).

As shown in Figures 7A–D, the DC vaccines result in the loss of naive T cells that differentiate into early effector T cells, which show a quick increase after the DC stimulation. The early effector cells gradually differentiate into short-lived effector T cells and memory T cells, both of which saturate at high levels. Compared to the normal DC vaccine, the caIKK-DCs increase the levels of short-lived effector and memory T cells by about 7-fold, demonstrating the enhanced immunogenic potency of the caIKK-DCs. At the molecular level, such improved immunogenic potency is caused by the upregulated activation of NF-κB pathway by IKKβ in DCs (Figures 7E,F).

**FIGURE 8:**
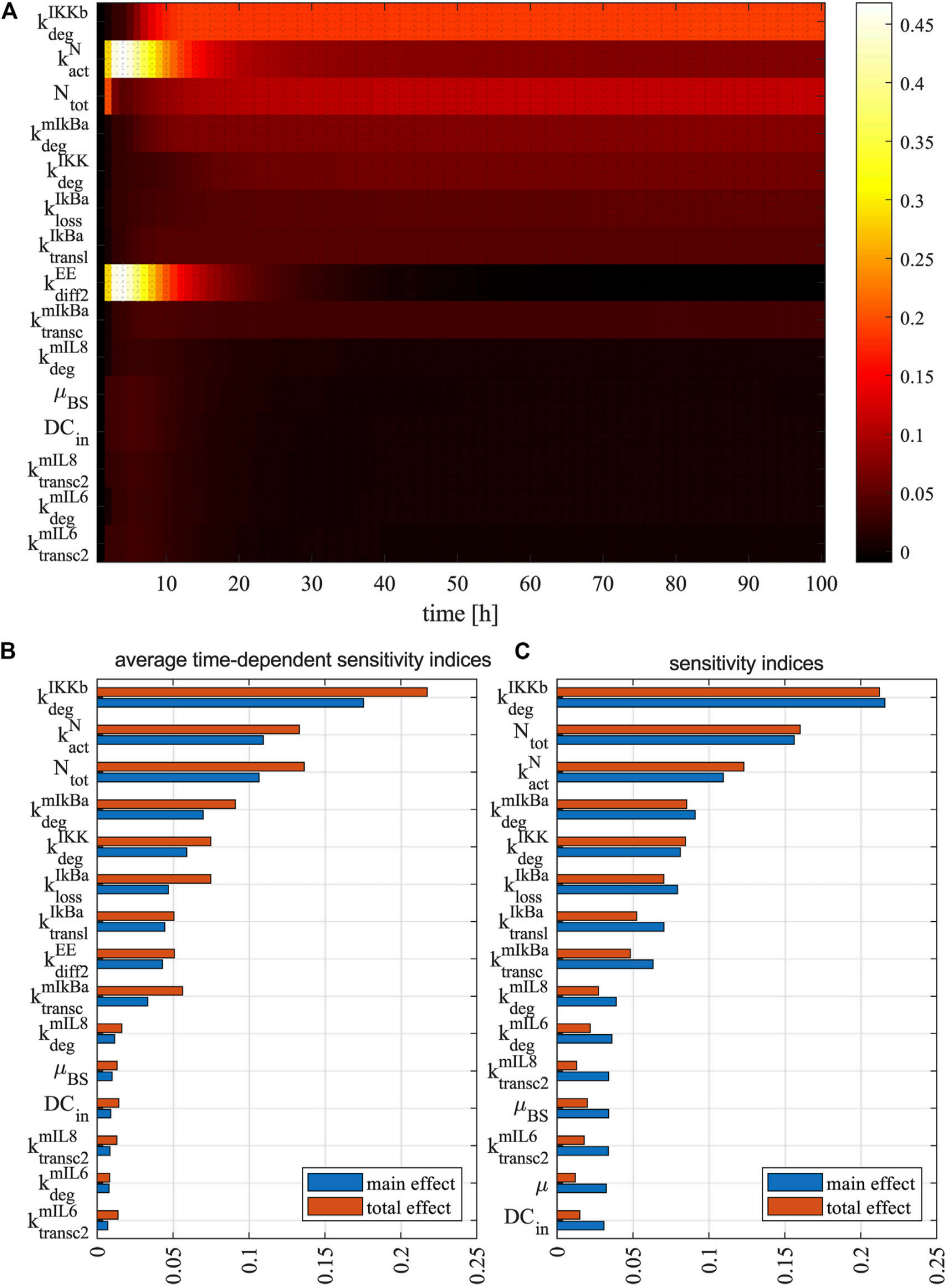
Sensitivity analysis of model parameters. **(A)** The heat map shows the time-dependent sensitivity indices of model parameters. The bar plots show **(B)** average time-dependent sensitivity indices of model parameters and **(C)** sensitivity indices of model parameters to the total amount of memory T cells. The main effect (blue bar) measures the direct contribution from an individual parameter to the model variable, while the total effect (red bar) measures the overall contribution including direct contribution and the amplification of the direct contribution due to interaction with all model parameters. Each graph shows the result for the most 15 sensitive parameters. The sensitivity indices of all parameters can be found in Supplementary Table S4.

Successful activation of naive T cells depends mainly on successful and strong interaction with antigen-presenting DCs (Timmerman and Levy, 1999, Abbas et al., 2018). The goal of a DC vaccine is to increase the number of the resulting memory T cells that contribute to a rapid immune response upon reactivation and form a long-lasting immunity (Akondy et al., 2017, Ando et al., 2019, Ahmed und Gray, 1996). Therefore, we performed sensitivity analyses to investigate the molecular mechanisms that are crucial for regulating the differentiation from naive T cells into early effectors and thus into memory T cells. Specifically, we used the global sensitivity method Sobol to compute sensitivities of model parameters to the population of memory T cells over the simulation time interval [0, 200] h (*see* Material and Methods). As shown in Figures 8A,B, the top-ranking 15 parameters show similar patterns in their sensitivity indices – the values are low shortly after the DC stimulation, gradually increase to a higher level, and stays at the high level until the end of the simulations. Among the top-ranking parameters, the most influential ones on the production of memory T cells are $k_{deg}^{IKKb}$ , $k_{act}^{N}$ , and *N*_*tot*_ that account for the degradation rate of IKKβ, the activation rate of naive T cells, and the total amount of free NF-κB. The less influential parameters are those associated with the degradation rate of IκBα mRNA ( $k_{mRNA}^{mIkBa}$ ), the degradation rate of the IKK protein ( $k_{deg}^{IKK},$ short-lived T-cell differentiation ( $k_{diff2}^{EE}$ ), loss of free IκBα ( $k_{loss}^{IkBa}$ ), and the production of IκBα mRNA and protein ( $k_{transc}^{mIkBa}$ and $k_{tranl}^{IkBa}$ ). The least influential parameters are the degradation rate and NF-κB-mediated transcription rate of IL-8 mRNA ( $k_{deg}^{mIL8}$ and $k_{transc2}^{mIL8}$ ), the degradation rate and NF-κB-mediated transcription rate of IL-6 mRNA ( $k_{deg}^{mIL6}$ and $k_{transc2}^{mIL6}$ ), the injected number of DCs (*DC*_*in*_), and the homing rate of DCs into spleen ( $\mu_{BS}$ ). We obtained similar results while computing the sensitivities of model parameters to the total amount of memory T cells (computed by taking the integral over the simulation interval [0, 200] h) (Figure 8C). The only difference is that the DC emigration rate from blood to other organs (*µ*) replaces $k_{diff2}^{EE}$ and appears as the least influencing parameters.

Further analysis showed that the length of simulation time and the variation of estimated parameter values have limited effects on model parameters’ sensitivity indices for the total amount of memory T cells over the simulation interval. Specifically, after extending the simulation time to 500 h, the most influential parameters (i.e., $k_{deg}^{IKKb}$ , $N_{tot}$ , $k_{act}^{N}$ , $k_{deg}^{mIkBa}$ , and $k_{deg}^{IKK}$ ) remain unchanged (Supplementary Table S5). In longer stimulation time, several parameters (i.e., *µ*, *µ*_*BS*_, *DC*_*in*_, $k_{tranl}^{IkBa}$ , $k_{deg}^{mIL6}$ and $k_{transc2}^{mIL6}$ ) become less influential and two parameters $k_{deg}^{IL6}$ (the degradation rate of IL-6) and *K*_*4*_ become more influential. After increasing parameter variations to 90% of their estimated values, most of the top 15 parameters remain in the list but have different ranking (Supplementary Table S6). The exceptions are *µ*_*BS*_, $k_{transc2}^{mIL6}$ , and $k_{deg}^{mIL8}$ that become less influential and drop out of the top 15 parameters. Besides, $k_{deg}^{TRAF2p}$ (degradation rate of TRAF2), $k_{deg}^{IL8}$ , and $k_{diff2}^{EE}$ (differentiation rate of early effector T cells into memory T cells) become more influential and are new top 15 parameters.

Taken together, the results demonstrated the ability of the multi-scale model to differentiate the ability of different DC vaccines to stimulate a T-cell response and to reveal the molecular mechanisms that are important for CD8^+^ T-cell activation through sensitivity analysis.

### *In Silico* Experiments to Predict the Effects of Modulation of Selected Molecules on DC-Mediated T-Cell Responses

**FIGURE 9:**
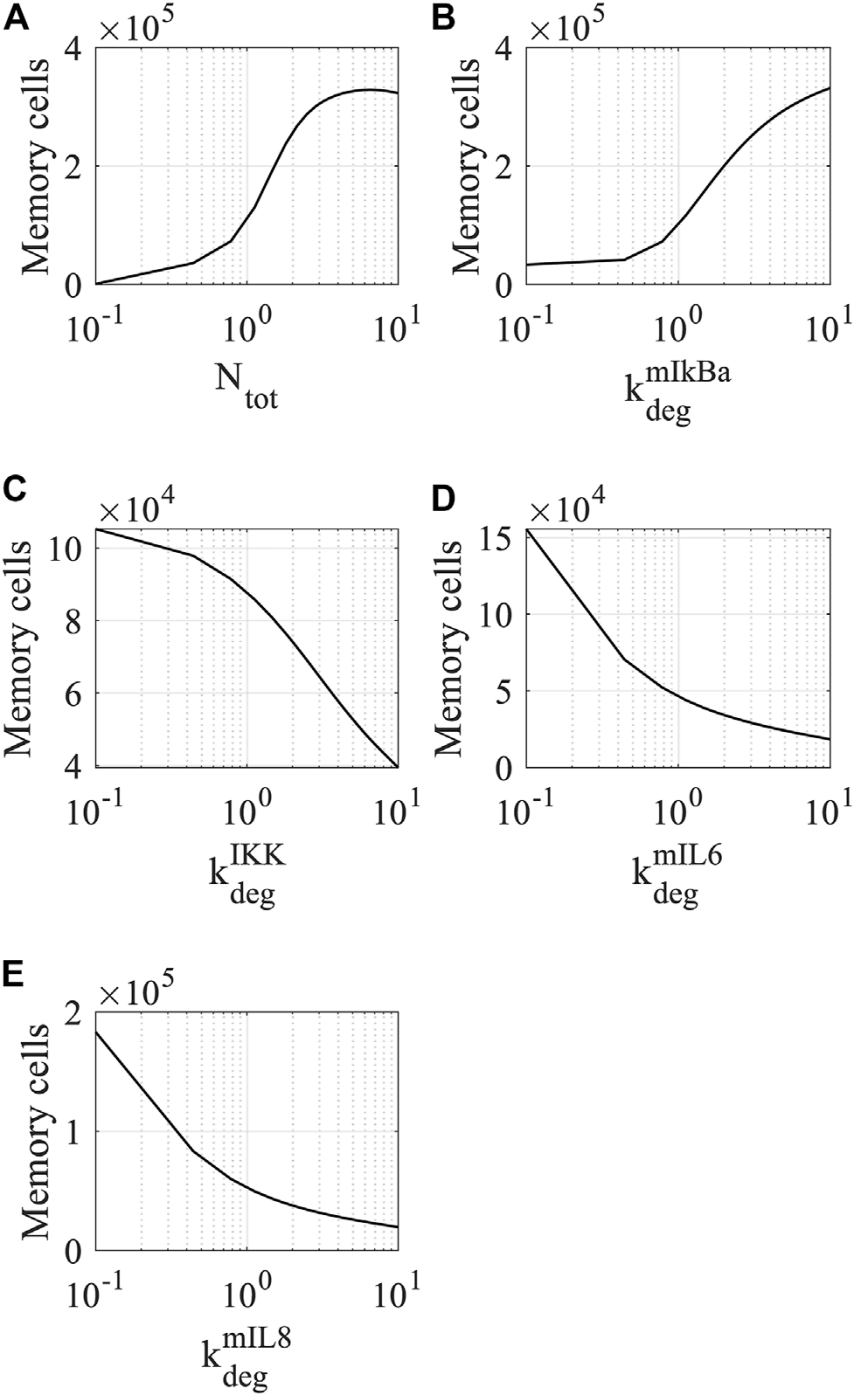
Simulations of memory T-cell dynamics by perturbing the values of sensitive parameters. The response of memory T cells to the modulation of **(A)** total amount of NF-κB, **(B)** degradation rate of IκBα mRNA, **(C)** degradation rate of IKK, **(D)** degradation rate of IL-6 mRNA, and **(E)** IL-8 mRNA. The steady state of memory T cells was determined at *t* = 4000 h. Each parameter was perturbed within the interval (the estimated value × [0.1, 10]).

After identifying influential parameters that can modulate the production of memory T cells, we ran simulations to predict how corresponding biological processes can change the dynamics of the memory T-cell population. From the 15 most influential parameters, we have selected five corresponding to specific genes that could be experimentally manipulated. Specifically, we perturbed those parameters in an interval that decreases and increases their estimated values by 10 folds (i.e., the estimated value × [0.1, 10]) and computed the steady state of memory T cells. As shown in Figure 9, the total amount of free NF-κB (*N*_*tot*_) and the degradation rate of IκBα mRNA ( $k_{deg}^{mIkBa}$ ) can positively affect the population of memory T cells. Decreasing the value of *N*_*tot*_ by 90% eliminates the T memory cells while increasing its value leads to an increased cell population. The cell population peaks when the value of *N*_*tot*_ increases by about 3–4 folds and slightly decreases when *N*_*tot*_ is at its maximum level. Such a phenomenon could be explained by the negative feedback loop formed by NF-κB and IκBα. Free NF-κB activates the transcription of IκBα, whose encoding protein reduces free NF-κB by forming complexes. Thus, when the level of IκBα protein reaches a certain threshold, free NF-κB starts decreasing leading to a reduced number of T cells. In contrast, the decreasing of $k_{deg}^{mIkBa}$ by 90% results in a slight reduction of T memory cells, and a 10-fold upregulation in the parameter value leads to about a 3-fold increase in the cell population. Biologically, the IκBα protein is an inhibitor of NF-κB and traps free NF-κB through forming complexes (Hayden and Matthew, 2008), so decreasing the IκBα mRNA results in the reduced level of the protein, thereby releasing more free NF-κB in DCs that is required for T-cell activation.

On the other hand, perturbation of the degradation rates of IKKβ ( $k_{deg}^{IKKb}$ ), IL-6 mRNA ( $k_{deg}^{mIL6}$ ), and IL-8 mRNA ( $k_{deg}^{mIL8}$ ) negatively affect the memory T-cell population. The dynamics of the cell population show similar patterns when the three parameters are perturbed in the specified interval – memory T cells are at the maximum level when the values of the parameters are reduced by 90% and at the minimum level when the values of the parameters increase by 10-folds. Biologically, IKKβ induces degradation of IκBα through phosphorylation (Hayden and Matthew, 2008), thereby releasing NF-κB from the complexes. Therefore, increasing the degradation of IKKβ leads to downregulation of NF-κB in DCs that reduce the induction of memory T cells. IL-6 and IL-8 secreted by DCs are required for differentiation of naive T cells into early effector T cells that further differentiate into short-lived effector T cells and memory T cells (Hunter and Jones, 2017, Taub et al., 1996), thereby increasing the degradation of the cytokines results in the decreased level of memory T cells.

**FIGURE 10:**
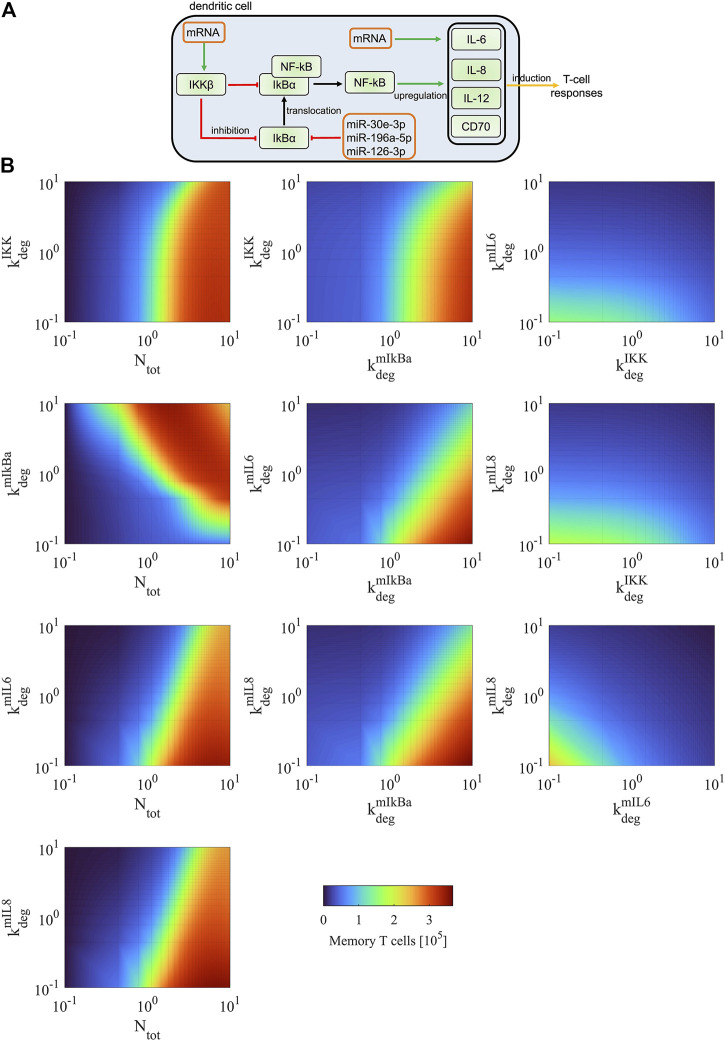
Model predictions of DC-mediated T-cell responses. **(A)** Scheme of experimental strategies to increase T-cell responses. The manipulation of the identified molecules includes upregulation of IKKβ and cytokines using transfected mRNAs encoding the corresponding proteins and downregulation of IκBα using transfected microRNAs. It has been shown that miR-30e, miR-196a, and miR-126 can repress the expression of IκBα (Jiang et al., 2012, Huang et al., 2014, Feng et al., 2012). **(B)** We simulated the dynamics of memory T cells for simultaneous modifying the values of two combined parameters. The number of memory T cells was determined at its steady state (*t* = 4000 h). Each parameter was perturbed within the interval (the estimated value × [0.1, 10]).

Furthermore, we simulated how the population of memory T cells changes when combining two parameters and perturbing them simultaneously. We are particularly interested in modulating the expression levels of genes that can be manipulated in DCs and thereby improving the immunogenic potency of DC vaccines (Figure 10A). For instance, one can upregulate the expression level of NF-κB through electroporating DCs with mRNA encoding constitutively active IKKβ. In addition, one could downregulate IκBα using microRNAs that repress gene expression at the post-transcriptional level to increase the level of NF-κB in DCs. One could also increase the levels of DC-secreted cytokines (such as IL-6 and IL-8) that are involved in the T-cell response through electroporation of the corresponding mRNAs into DCs.

As shown in Figure 10B left column, directly increasing the expression of free NF-κB shows dominant effects on the upregulation of memory T cells for any combination with other parameters. However, this manipulation is experimentally difficult, as NF-κB is a protein complex and requires the presence of subunits and heterodimerization to be functional. Alternatively, it is experimentally achievable by manipulating the expression of NF-κB regulators. Simultaneously modulating the expression of IKKβ (upregulation) and IκBα (downregulation) can result in an effective increase of memory T cells (Figure 10B middle column). Modulating the expression of IκBα has stronger effects than modulating IKKβ. Compared to single modulation of the NF-κB regulators, the combined modulation increases the population of memory T cells by 80–120%. Modulating the levels of IκBα together with DC-secreted cytokines (IL-6 and IL-8) is also an effective regulation of memory T cells but shows different dynamics compared to the modulation of both NF-κB regulators (i.e., IKKβ and IκBα) (Figure 10B middle column). The cytokines are less influential than IκBα in regulating the T-cell response, as modulating IκBα directly affects the expression of free NF-κB that can regulate the expression of multiple cytokines and membrane proteins (i.e., IL-6, IL-8, IL-12, and CD70) involved in T-cell activation. When we manipulated the expression level of IKKβ with a cytokine, the effects on increasing memory T cells are mild (Figure 10B right column). When both cytokines were simultaneously modulated, the effect on increasing memory T cells is also limited. This is due to the reason that T-cell activation also depends on other proteins (i.e., IL-12 and CD70), and their unchanged levels act as a limiting factor in the increase of memory T cells. This is in line with findings in a biological model system examining CTL-priming and memory formation in the presence or absence of T-cell help, where it was shown that direct cell-cell contact was crucial and soluble factors were not sufficient (Hoyer et al., 2014). Taken together, the simulations predicted that combined manipulation of IκBα and cytokines is an efficient strategy for increasing memory T cells in experiments, and such manipulation shows better performance than manipulating the expression levels of only the NF-κB regulators or the cytokines.

## Discussion

Model derivation. In this work, we developed a multi-level model to study DC-based anti-cancer immunotherapy. The model considers three spatiotemporal, different but interlinked stages. The first stage models the bio-distribution of the intravenously injected maturated DCs into key organs of the human body including the lung, liver, and spleen. They are used as a representative immune organ to which DCs are trafficked. Except for intravenous injection, there are other clinical administrations of DC vaccine such as intra-lymph node injection and subcutaneous injection. However, from the physiological point of view, the immune response triggered by DCs may be similar (Mackensen et al., 1999). The rationale to include this mechanism was to achieve a precise quantitative description of how DCs get distributed between organs, how many DCs reach the spleen, and how long DCs remain in the spleen for stimulating CTL response (Ludewig et al., 2004, Eggert et al., 1999). The second stage of the model accounts for the intracellular processes responsible for the *in vitro* maturation of DCs. This activation is induced by the activation of signaling pathways through ligands like TNFα, IL-1β, or LPS. Then, matured DCs secret cytokines (such as IL-6, IL-8, and IL-12) that are involved in the stimulation of a CTL response. It was important to include this level in the model because our aim was to investigate computationally the effect of molecular modulation of key regulatory pathways. In the last stage, the model simulates interactions between DCs and naive spleen resident T cells. The equations used account for the differentiation of T cells into early effector cells and the transformation of T cells into either short-lived effector cells or memory cells. Specifically, we simulated the effect of tumor antigen presentation, key cytokines secretions, and surface protein markers expression in the interaction of DC and T cells, and therefore the molecular and cell-cell communication are interlinked in the model. The amount of memory T cells is used as an indicator of the effectiveness of the DC-based immunotherapy.

In the literature, there are serval models devoted to understanding DC-mediated T-cell response in the context of immune response or immunotherapy. Most of them have a focus on only the cell populations’ interactions and dynamics. For instance, Bianca et al. used ODEs to account for the different stages of the therapy including vaccination, immune cells, and tumor cells. The model includes the dynamics of both humoral and cellular immune responses to associated tumor antigens. The goal was to investigate different vaccination protocols using sensitivity analyses to improve treatment (Bianca et al., 2012). Ludewig et al. (2004) modeled DC distribution after vaccination to determine key parameters that control interactions between DCs and T cells. Furthermore, Serre et al. developed a mathematical model accounting for a cancer treatment combining immunotherapy with radiotherapy. The author used the model to simulate the primary and secondary (or memory) immune response induced by the combined therapy and proposed an optimal schedule for the therapy (Serre et al., 2016). By modeling the interactions between DCs with different types of T cells, Arabameri et al., (2018) showed an optimal configuration of DC vaccine to strengthen DC-T cell interactions, and therefore efficiently reducing tumor size. Castillo-Montiel et al. developed a model with delay differential equations to study cellular mechanisms of DC-based immunotherapy for melanoma. The authors showed the power of the model in reproducing data from experimental trials and predicting possible protocols to improve the immunotherapy while producing them in labs (Castillo-Montiel et al., 2015). Our model not only considers interactions between DCs and T cells but also the effect of signaling pathways governing the triggering of phenotypic changes in DCs, which underlie the efficacy of the CTL response. Such a multi-level model allows for the identification of molecular targets and other therapy parameters (such as vaccination schedules and DC dosage) that can be experimentally manipulated to improve the effectiveness of the therapy. In the future, the model can be expanded by considering the interactions between immune cells and tumor cells, such as the regulatory role of checkpoint proteins (e.g., CTLA-4 and PD-1) on T cell activation. This expansion will make the model suitable for studying the dynamics of immune-tumor interactions in the tumor microenvironment.

Model calibration. To calibrate the multi-level model, we used different data sets accounting for the three stages of the DC immunotherapy because in the literature, we could not find a single, comprehensive data set that measures the dynamics of all different aspects of the DC vaccination. Furthermore, obtaining new data for some of the processes in the model is currently very challenging due to ethical considerations. For example, the data on DC bio-distribution in humans utilized in the model could not be generated *de novo* with the current ethical rules, at least in Europe. An alternative is to calibrate the model utilizing mouse data, but we think the animal data could compromise the precision of cell dynamics such as the timing of DC bio-distribution. To circumvent these issues, we selected consistent data sets that represent the dynamics of the DC vaccination at different levels. In such a manner, the data complement each other to characterize the model at all scales.

While performing parameter optimization, we combined global and local optimization methods and fitted different parts of the model to the corresponding data set separately. This strategy is widely used by the community to deal with complex and large systems whose objective functions are usually multi-modal and non-convex (Villaverde et al., 2019). Alternatively, one can perform parameter optimization using a multi-starting strategy that locally searches for the parameter space from different starting points (Raue et al., 2013). However, such a strategy becomes time-consuming when a model contains many parameters because to ensure reasonable coverage of the parameter space by local searches, the number of starting points will increase exponentially. A solution for such an issue is utilizing parallel computing that can significantly increase the computation efficacy on a high-performance computing cluster (Penas et al., 2017).

The hybrid approach (i.e., global pattern search followed by a gradient-based method) for ensures that the cost function for parameter estimation is not trapped in local minima. The best parameter estimates with the minimum cost function were used to assess practical identifiability of estimated parameters. The analysis showed that a few parameters in the NF-κB pathway underlying DC maturation are practically unidentifiable. Such uncertainties are most likely due to the lack of experimental data and the small variances of the estimated parameter values imply limited impacts on their biological interpretations. More advanced methods are available for analyzing the practical identifiability of estimated parameters (Fröhlich et al., 2014). For instance, the profile likelihood approach perturbs the value of a parameter in a small interval while keeping the other parameters unchanged to draw a profile of the cost function. The shape of the profile is used to identify whether or not the parameter is identifiable (Flassig and Sundmacher, 2012). In a fully clinic-oriented setup for model calibration and to solve the practical identifiability issue of parameters, one can produce additional experimental data for parameter estimation, simplify the model by reducing parameters, or fix the values of unidentifiable parameters using prior knowledge (Villaverde et al., 2021).

Sensitivity analyses to detect key parameters and processes. We performed sensitivity analyses to identify model parameters that are crucial to biological processes. Such a method has been widely used to evaluate the influence of model parameters (e.g., kinetic rate constants) on model outputs (e.g., the steady states of model variables) (Zi, 2011, Nikolov et al., 2010). Depending on strategies used for perturbing model parameters, sensitivity analysis can be classified into local and global analysis. Local sensitivity analysis provides a description of the behavior near a specified operating condition, whereas global sensitivity analysis uses wide ranges of parameter spaces and addresses global behavior of model parameters using statistical methods (Frey and Patil, 2002). We used the Sobol method for performing sensitivity analysis, but there are other methods for computing sensitivity coefficients based on rank transforms (such as partial rank regression coefficient) that show better performance on nonlinear and non-monotonic models (Frey and Patil, 2002). Using both local and global sensitivity analysis, our data showed consistent results in identifying sensitive parameters for affecting the population of memory T cells, implying the significant impact of the corresponding molecules on CTL responses. The results indicated that intracellular or intercellular processes (i.e., DC bio-distribution or DC-T cell interaction) influence the efficacy of DC vaccination at inducing memory T cells. Since others investigated cell-cell interactions (*see* (DePillis et al., 2013) for example.), we focused on the wiring of the intracellular DC circuits. The simulations suggested that the activity of several proteins belonging to the network can affect the therapy effectiveness in terms of memory T cell induction.

Predictive model simulations. Our simulations showed that the perturbation of NF-κB and its regulators (i.e., IKKβ and IκBα) have a strong impact on the population of memory T cells. This suggested that enhanced and long-lasting activation of the NF-κB pathway is particularly effective in improving the immunogenic potency of DCs. Since our model accounts for the effect of known negative feedback loops regulating NF-κB activation, the model predictions point to strategies that can help in circumventing the detrimental effect of these loops. However, only improving the population of effective T cells may not be enough to ensure the long-term survival of cancer patients, as T cell subsets and heterogeneity of T cell states in tumors also play a major role in mediating immunotherapy responses (Philip and Schietinger, 2021). In addition, we showed that the cytokines (i.e., IL-6 and IL-8), necessary for the efficient activation of T-cell responses, are also influential on the effectiveness for the DC immunotherapy. It is also worth noting that IL-12 is another important immunostimulatory cytokine and incorporation or endogenous induction of this cytokine is shown to consistently benefit DC-based immunotherapy (Brussel et al., 2012). Furthermore, the production of cytokines by DCs depends not only on the activation of the NF-κB pathway but also on the methods used for isolating human monocytes that can differentiate into DCs (Elkord et al., 2005).

In the current version of the model, the intracellular signaling module is centered on the activation of the NF-κB signaling pathway, which is known to be pivotal in the maturation and activation of DCs. However, other regulatory pathways also play an important role in DC vaccination, and these pathways can crosstalk with each other forming a large regulatory network (Lai et al., 2021). Including these pathways into an intracellular module requires access to time-series data of their activation in DCs. Alternatively, they could be transformed into a Boolean or multi-logic model reproducing the wiring of the network as shown by others in the context of cancer (Khan et al., 2017) and immunity (Saez-Rodriguez et al., 2007). One could also build a hybrid model by combining ODE and Boolean modeling. The ODE model accounts for the core regulatory circuit around NF-κB and the Boolean model for genes and signaling proteins not belonging to the core circuit (Khan et al., 2014). Similarly, one could add spatial details into the interactions between DCs and T cells in the spleen. To do so, one has to develop models in partial differential equations or use agent-based models. Both types of models require detailed spatial information like the one provided by *in vivo* imaging. This is doable and has been implemented in mouse models for characterizing DC-T cell interactions in the lymph nodes (Celli et al., 2012).

Taken together, we demonstrated the potential of our multi-level model in tackling the complexity of DC-based immunotherapy and identifying potential molecules for improving its effectiveness. Besides, we believe such an approach is adaptable and applicable to optimize other cell-based cancer immunotherapies like CAR-T cells.

## Data Availability

The code used for performing model simulations and analyses in this work is accessible at https://doi.org/10.5281/zenodo.5744196. Further details and other data that support the findings of our study are available from the corresponding author upon request.

## References

[B1] Abbas A. K. Lichtman A. H. Pillai S. (2018). Cellular and Molecular Immunology, 9. Philadelphia: Elsevier.

[B2] Ahmed R. Gray D. (1996). Immunological Memory and Protective Immunity: Understanding Their Relation. Science 272, 54–60. 10.1126/science.272.5258.54 8600537

[B3] Akondy R. S. Fitch M. Edupuganti S. Yang S. Kissick H. T. Li K. W. (2017). Origin and Differentiation of Human Memory CD8 T Cells After Vaccination. Nature 552, 362–367. 10.1038/nature24633 29236685 PMC6037316

[B4] Ando M. Ito M. Srirat T. Kondo T. Yoshimura A. (2019). Memory T Cell, Exhaustion, and Tumor Immunity. Immunological Med. 43, 1–9. 10.1080/25785826.2019.1698261 31822213

[B5] Arabameri A. Asemani D. Hajati J. (2018). Mathematical Modeling of In-vivo Tumor-Immune Interactions for the Cancer Immunotherapy Using Matured Dendritic Cells. J. Biol. Syst. 26, 167–188. 10.1142/s0218339018500080

[B6] Arulraj T. Barik D. (2018). Mathematical Modeling Identifies Lck as a Potential Mediator for PD-1 Induced Inhibition of Early TCR Signaling. PLoS ONE 13, e0206232. 10.1371/journal.pone.0206232 30356330 PMC6200280

[B7] Badovinac V. P. Porter B. B. Harty J. T. (2002). Programmed Contraction of CD8+ T Cells After Infection. Nat. Immunol. 3, 619–626. 10.1038/ni804 12055624

[B8] Barinov A. Galgano A. Krenn G. Tanchot C. Vasseur F. Rocha B. (2017). CD4/CD8/Dendritic Cell Complexes in the Spleen: CD8+ T Cells Can Directly Bind CD4+ T Cells and Modulate Their Response. PLoS ONE 12, e0180644. 10.1371/journal.pone.0180644 28686740 PMC5501581

[B9] Bianca C. Chiacchio F. Pappalardo F. Pennisi M. (2012). Mathematical Modeling of the Immune System Recognition to Mammary Carcinoma Antigen. BMC Bioinformatics 13, S21. 10.1186/1471-2105-13-s17-s21 PMC3521211 23281916

[B10] Bode K. A. Schmitz F. Vargas L. Heeg K. Dalpke A. H. (2009). Kinetic of RelA Activation Controls Magnitude of TLR-Mediated IL-12p40 Induction. J. Immunol. 182, 2176–2184. 10.4049/jimmunol.0802560 19201871

[B11] Brossart P. Wirths S. Brugger W. Kanz L. (2001). Dendritic Cells in Cancer Vaccines. Exp. Hematol. 29, 1247–1255. 10.1016/s0301-472x(01)00730-5 11698120

[B12] Brussel I. V. Berneman Z. N. Cools N. (2012). Optimizing Dendritic Cell-Based Immunotherapy: Tackling the Complexity of Different Arms of the Immune System. Mediators Inflamm. 2012, 1–14. 10.1155/2012/690643 PMC3407661 22851815

[B13] Bullock T. N. J. Mullins D. W. Engelhard V. H. (2003). Antigen Density Presented by Dendritic Cells In Vivo Differentially Affects the Number and Avidity of Primary, Memory, and Recall CD8+ T Cells. J. Immunol. 170, 1822–1829. 10.4049/jimmunol.170.4.1822 12574347

[B14] Castiglione F. Piccoli B. (2007). Cancer Immunotherapy, Mathematical Modeling and Optimal Control. J. Theor. Biol. 247, 723–732. 10.1016/j.jtbi.2007.04.003 17543345

[B15] Castillo-Montiel E. Chimal-Eguía J. C. Tello J. I. Piñon-Zaráte G. Herrera-Enríquez M. Castell-Rodríguez A. (2015). Enhancing Dendritic Cell Immunotherapy for Melanoma Using a Simple Mathematical Model. Theor. Biol. Med. Model. 12, 11. 10.1186/s12976-015-0007-0 26054860 PMC4469008

[B16] Celli S. Day M. Müller A. J. Molina-Paris C. Lythe G. Bousso P. (2012). How many Dendritic Cells Are Required to Initiate a T-Cell Response? Blood 120, 3945–3948. 10.1182/blood-2012-01-408260 22995897

[B17] Cess C. G. Finley S. D. (2020). Multi-Scale Modeling of Macrophage-T Cell Interactions within the Tumor Microenvironment. Plos Comput. Biol. 16, e1008519. 10.1371/journal.pcbi.1008519 33362239 PMC7790427

[B18] Chis O.-T. Banga J. R. Balsa-Canto E. (2011). Structural Identifiability of Systems Biology Models: A Critical Comparison of Methods. PLoS ONE 6, e27755. 10.1371/journal.pone.0027755 22132135 PMC3222653

[B19] De Odorico I. Spaulding K. A. Pretorius D. H. Lev-Toaff A. S. Bailey T. B. Nelson T. R. (1999). Normal Splenic Volumes Estimated Using Three-Dimensional Ultrasonography. J. Ultrasound Med. 18, 231–236. 10.7863/jum.1999.18.3.231 10082358

[B20] DePillis L. Gallegos A. Radunskaya A. (2013). A Model of Dendritic Cell Therapy for Melanoma. Front. Oncol. 3, 56. 10.3389/fonc.2013.00056 23516248 PMC3601335

[B21] Dudley M. E. Wunderlich J. R. Robbins P. F. Yang J. C. Hwu P. Schwartzentruber D. J. (2002). Cancer Regression and Autoimmunity in Patients After Clonal Repopulation with Antitumor Lymphocytes. Science 298, 850–854. 10.1126/science.1076514 12242449 PMC1764179

[B22] Eggert A. A. Schreurs M. W. Boerman O. C. Oyen W. J. de Boer A. J. Punt C. J. (1999). Biodistribution and Vaccine Efficiency of Murine Dendritic Cells Are Dependent on the Route of Administration. Cancer Res. 59 (14), 3340–3345. 10416590

[B23] Elkord E. Williams P. E. Kynaston H. Rowbottom A. W. (2005). Human Monocyte Isolation Methods Influence Cytokine Production from In Vitro Generated Dendritic Cells. Immunology 114, 204–212. 10.1111/j.1365-2567.2004.02076.x 15667565 PMC1782075

[B24] Feng X. Wang H. Ye S. Guan J. Tan W. Cheng S. (2012). Up-Regulation of microRNA-126 May Contribute to Pathogenesis of Ulcerative Colitis via Regulating NF-kappaB Inhibitor IκBα. PloS One 7 (12), e52782. 10.1371/journal.pone.0052782 23285182 PMC3532399

[B25] Fey D. Halasz M. Dreidax D. Kennedy S. P. Hastings J. F. Rauch N. (2015). Signaling Pathway Models as Biomarkers: Patient-Specific Simulations of JNK Activity Predict the Survival of Neuroblastoma Patients. Sci. Signal. 8 (408), ra130. 10.1126/scisignal.aab0990 26696630

[B26] Flassig R. J. Sundmacher K. (2012). Optimal Design of Stimulus Experiments for Robust Discrimination of Biochemical Reaction Networks. Bioinformatics 28, 3089–3096. 10.1093/bioinformatics/bts585 23047554 PMC3516143

[B27] Fong L. Engleman E. G. (2000). Dendritic Cells in Cancer Immunotherapy. Annu. Rev. Immunol. 18, 245–273. 10.1146/annurev.immunol.18.1.245 10837059

[B28] Frey H. C. Patil S. R. (2002). Identification and Review of Sensitivity Analysis Methods. Risk Anal. 22, 553–578. 10.1111/0272-4332.00039 12088234

[B29] Fröhlich F. Theis F. J. Hasenauer J. (2014). “Uncertainty Analysis for Non-identifiable Dynamical Systems: Profile Likelihoods, Bootstrapping and More,” in Computational Methods in Systems Biology. CMSB 2014. Lecture Notes in Computer Science, Manchester, November 17–19, 2014. Editors Mendes P. Dada J. O. Smallbone K. (Cham: Springer), 61–72. 10.1007/978-3-319-12982-2_5

[B30] Gong C. Milberg O. Wang B. Vicini P. Narwal R. Roskos L. (2017). A Computational Multiscale Agent-Based Model for Simulating Spatio-Temporal Tumour Immune Response to PD1 and PDL1 Inhibition. J. R. Soc. Interf. 14, 20170320. 10.1098/rsif.2017.0320 PMC5636269 28931635

[B31] Hayden M. S. Ghosh S. (2008). Shared Principles in NF-Κb Signaling. Cell 132, 344–362. 10.1016/j.cell.2008.01.020 18267068

[B32] Henrickson S. E. Mempel T. R. Mazo I. B. Liu B. Artyomov M. N. Zheng H. (2008). T Cell Sensing of Antigen Dose Governs Interactive Behavior with Dendritic Cells and Sets a Threshold for T Cell Activation. Nat. Immunol. 9, 282–291. 10.1038/ni1559 18204450 PMC2698867

[B33] Hernandez A. Burger M. Blomberg B. B. Ross W. A. Gaynor J. J. Lindner I. (2007). Inhibition of NF-Κb During Human Dendritic Cell Differentiation Generates Anergy and Regulatory T-Cell Activity for One but Not Two Human Leukocyte Antigen DR Mismatches. Hum. Immunol. 68, 715–729. 10.1016/j.humimm.2007.05.010 17869645 PMC2245875

[B34] Hesse J. Martinelli J. Aboumanify O. Ballesta A. Relógio A. (2021). A Mathematical Model of the Circadian Clock and Drug Pharmacology to Optimize Irinotecan Administration Timing in Colorectal Cancer. Comput. Struct. Biotechnol. J. 19, 5170–5183. 10.1016/j.csbj.2021.08.051 34630937 PMC8477139

[B35] Hoyer S. Prommersberger S. Pfeiffer I. A. Schuler-Thurner B. Schuler G. Dörrie J. (2014). Concurrent Interaction of DCs with CD4+and CD8+T Cells Improves Secondary CTL Expansion: It Takes Three to Tango. Eur. J. Immunol. 44, 3543–3559. 10.1002/eji.201444477 25211552

[B36] Huang F. Tang J. Zhuang X. Zhuang Y. Cheng W. Chen W. (2014). MiR-196a Promotes Pancreatic Cancer Progression by Targeting Nuclear Factor Kappa-B-Inhibitor Alpha. PloS One 9 (2), e87897. 10.1371/journal.pone.0087897 24504166 PMC3913664

[B37] Hunter C. A. Jones S. A. (2017). IL-6 as a keystone Cytokine in Health and Disease. Nat. Immunol. 16, 448–457. 10.1038/ni.3153 25898198

[B38] Jiang L. Lin C. Song L. Wu J. Chen B. Ying Z. (2012). MicroRNA-30e* Promotes Human Glioma Cell Invasiveness in an Orthotopic Xenotransplantation Model by Disrupting the NF-κB/IκBα Negative Feedback Loop. J. Clin. Invest. 122 (1), 33–47. 10.1172/JCI58849 22156201 PMC3248293

[B39] Khan F. M. Marquardt S. Gupta S. K. Knoll S. Schmitz U. Spitschak A. (2017). Unraveling a Tumor Type-Specific Regulatory Core Underlying E2F1-Mediated Epithelial-Mesenchymal Transition to Predict Receptor Protein Signatures. Nat. Commun. 8 (1), 198. 10.1038/s41467-017-00268-2 28775339 PMC5543083

[B40] Khan F. M. Schmitz U. Nikolov S. Engelmann D. Pützer B. M. Wolkenhauer O. (2014). Hybrid Modeling of the Crosstalk Between Signaling and Transcriptional Networks Using Ordinary Differential Equations and Multi-Valued Logic. Biochim. Biophys. Acta (Bba) - Proteins Proteomics 1844, 289–298. 10.1016/j.bbapap.2013.05.007 23692959

[B41] Kogan Y. Halevi–Tobias K. Elishmereni M. Vuk-Pavlović S. Agur Z. (2012). Reconsidering the Paradigm of Cancer Immunotherapy by Computationally Aided Real-Time Personalization. Cancer Res. 72, 2218–2227. 10.1158/0008-5472.CAN-11-4166 22422938

[B42] Lai X. Dreyer F. S. Cantone M. Eberhardt M. Gerer K. F. Jaitly T. (2021). Network- and Systems-Based Re-Engineering of Dendritic Cells with Non-Coding RNAs for Cancer Immunotherapy. Theranostics 11, 1412–1428. 10.7150/thno.53092 33391542 PMC7738891

[B43] Ludewig B. Krebs P. Junt T. Metters H. Ford N. J. Anderson R. M. (2004). Determining Control Parameters for Dendritic Cell-Cytotoxic T Lymphocyte Interaction. Eur. J. Immunol. 34, 2407–2418. 10.1002/eji.200425085 15307173

[B44] Mackensen A. Krause T. Blum U. Uhrmeister P. Mertelsmann R. Lindemann A. (1999). Homing of Intravenously and Intralymphatically Injected Human Dendritic Cells Generated In Vitro from CD34 + Hematopoietic Progenitor Cells. Cancer Immunol. Immunother. 48, 118–122. 10.1007/s002620050555 10414465 PMC11037217

[B45] Michiels A. Tuyaerts S. Bonehill A. Corthals J. Breckpot K. Heirman C. (2005). Electroporation of Immature and Mature Dendritic Cells: Implications for Dendritic Cell-Based Vaccines. Gene Ther. 12, 772–782. 10.1038/sj.gt.33024710.1038/sj.gt.3302471 15750615

[B46] Morandi F. Chiesa S. Bocca P. Millo E. Salis A. Solari M. (2006). Tumor mRNA-Transfected Dendritic Cells Stimulate the Generation of CTL that Recognize Neuroblastoma-Associated Antigens, Kill Tumor Cells: Immunotherapeutic Implications. Neoplasia 8, 833–842. 10.1593/neo.06415 17032500 PMC1715922

[B47] Mueller S. N. Gebhardt T. Carbone F. R. Heath W. R. (2013). Memory T Cell Subsets, Migration Patterns, and Tissue Residence. Annu. Rev. Immunol. 31, 137–161. 10.1146/annurev-immunol-032712-095954 23215646

[B48] Nikolov S. Lai X. Liebal U. W. Wolkenhauer O. Vera J. (2010). Integration of Sensitivity and Bifurcation Analysis to Detect Critical Processes in a Model Combining Signalling and Cell Population Dynamics. Int. J. Syst. Sci. 41, 81–105. 10.1080/00207720903147746

[B49] Palucka K. Banchereau J. (2013). Dendritic-Cell-Based Therapeutic Cancer Vaccines. Immunity 39, 38–48. 10.1016/j.immuni.2013.07.004 23890062 PMC3788678

[B50] Penas D. R. González P. Egea J. A. Doallo R. Banga J. R. (2017). Parameter Estimation in Large-Scale Systems Biology Models: A Parallel and Self-Adaptive Cooperative Strategy. BMC Bioinformatics 18 (1), 52. 10.1186/s12859-016-1452-4 28109249 PMC5251293

[B51] Pfeiffer I. A. Hoyer S. Gerer K. F. Voll R. E. Knippertz I. Gückel E. (2014). Triggering of NF-Κb in Cytokine-Matured Human DCs Generates Superior DCs for T-Cell Priming in Cancer Immunotherapy. Eur. J. Immunol. 44, 3413–3428. 10.1002/eji.201344417 25100611

[B52] Philip M. Schietinger A. (2021). CD8+ T Cell Differentiation and Dysfunction in Cancer. Nat. Rev. Immunol. [Epub ahead of print]. 10.1038/s41577-021-00574-3 PMC9792152 34253904

[B53] Pianosi F. Beven K. Freer J. Hall J. W. Rougier J. Stephenson D. B. (2016). Sensitivity Analysis of Environmental Models: A Systematic Review with Practical Workflow. Environ. Model. Softw. 79, 214–232. 10.1016/j.envsoft.2016.02.008

[B54] Raue A. Schilling M. Bachmann J. Matteson A. Schelke M. Kaschek D. (2013). Lessons Learned from Quantitative Dynamical Modelingin Systems Biology. PLoS One 8 (9), e74335. 10.1371/journal.pone.0074335 24098642 PMC3787051

[B55] Raue A. Kreutz C. Maiwald T. Bachmann J. Schilling M. Klingmüller U. (2009). Structural and Practical Identifiability Analysis of Partially Observed Dynamical Models by Exploiting the Profile Likelihood. Bioinformatics 25, 1923–1929. 10.1093/bioinformatics/btp358 19505944

[B56] Saez-Rodriguez J. Simeoni L. Lindquist J. A. Hemenway R. Bommhardt U. Arndt B. (2007). A Logical Model Provides Insights into T Cell Receptor Signaling. Plos Comput. Biol. 3, e163. 10.1371/journal.pcbi.0030163 17722974 PMC1950951

[B57] Saltelli A. Ratto M. Andres T. Campolongo F. Cariboni J. Gatelli D. (2008). Global Sensitivity Analysis: The Primer, 3. Chichester, United Kingdom: Wiley-Interscience.

[B58] Santos G. Nikolov S. Lai X. Eberhardt M. Dreyer F. S. Paul S. (2016). Model-Based Genotype-Phenotype Mapping Used to Investigate Gene Signatures of Immune Sensitivity and Resistance in Melanoma Micrometastasis. Sci. Rep. 6, 24967. 10.1038/srep24967 27113331 PMC4844979

[B59] Sarrazin F. Pianosi F. Wagener T. (2016). Global Sensitivity Analysis of Environmental Models: Convergence and Validation. Environ. Model. Softw. 79, 135–152. 10.1016/j.envsoft.2016.02.005

[B60] Schaft N. Wellner V. Wohn C. Schuler G. Dörrie J. (2013). CD8+ T-Cell Priming and Boosting: More Antigen-Presenting DC, or More Antigen Per DC? Cancer Immunol. Immunother. 62, 1769–1780. 10.1007/s00262-013-1481-z 24114143 PMC11029756

[B61] Schreurs M. W. Eggert A. A. de Boer A. J. Vissers J. L. van Hall T. Offringa R. (2000). Dendritic Cells Break Tolerance and Induce Protective Immunity against a Melanocyte Differentiation Antigen in an Autologous Melanoma Model. Cancer Res. 60, 6995–7001. 11156402

[B62] Schulz C. Lai X. Bertrams W. Jung A. L. Sittka-Stark A. Herkt C. E. (2017). THP-1-Derived Macrophages Render Lung Epithelial Cells Hypo-Responsive to *Legionella P* - a Systems Biology Study. Sci. Rep. 7 (1), 11988. 10.1038/s41598-017-12154-4 28931863 PMC5607273

[B63] Serre R. Benzekry S. Padovani L. Meille C. André N. Ciccolini J. (2016). Mathematical Modeling of Cancer Immunotherapy and its Synergy with Radiotherapy. Cancer Res. 76, 4931–4940. 10.1158/0008-5472.can-15-3567 27302167

[B64] Sobotta S. Raue A. Huang X. Vanlier J. Jünger A. Bohl S. (2017). Model Based Targeting of IL-6-Induced Inflammatory Responses in Cultured Primary Hepatocytes to Improve Application of the JAK Inhibitor Ruxolitinib. Front. Physiol. 8, 775. 10.3389/fphys.2017.00775 29062282 PMC5640784

[B65] Sprooten J. Ceusters J. Coosemans A. Agostinis P. De Vleeschouwer S. Zitvogel L. (2019). Trial Watch: Dendritic Cell Vaccination for Cancer Immunotherapy. OncoImmunology 8, 1638212. 10.1080/2162402x.2019.1638212 PMC6791419 31646087

[B66] Steinman R. M. (1989). Dendritic Cells: Clinical Aspects. Res. Immunol. 140, 911–918. 10.1016/0923-2494(89)90054-0 2697915

[B67] Tang B. (1993). Orthogonal Array-Based Latin Hypercubes. J. Am. Stat. Assoc. 88, 1392–1397. 10.1080/01621459.1993.10476423

[B68] Tas S. W. de Jongde Jong E. C. Hajji N. May M. J. Ghosh S. Vervoordeldonk M. J. (2005). Selective Inhibition of NF-kappaB in Dendritic Cells by the NEMO-Binding Domain Peptide Blocks Maturation and Prevents T Cell Proliferation and Polarization. Eur. J. Immunol. 35, 1164–1174. 10.1002/eji.200425956 15770694

[B69] Taub D. D. Anver M. Oppenheim J. J. Longo D. L. Murphy W. J. (1996). T Lymphocyte Recruitment by Interleukin-8 (IL-8). IL-8-Induced Degranulation of Neutrophils Releases Potent Chemoattractants for Human T Lymphocytes Both In Vitro and In Vivo. J. Clin. Invest. 97, 1931–1941. 10.1172/JCI118625 8621778 PMC507263

[B70] Timmerman J. M. Levy R. (1999). Dendritic Cell Vaccines for Cancer Immunotherapy. Annu. Rev. Med. 50, 507–529. 10.1146/annurev.med.50.1.507 10073291

[B71] Vera J. Schmitz U. Lai X. Engelmann D. Khan F. M. Wolkenhauer O. (2013). Kinetic Modeling-Based Detection of Genetic Signatures that Provide Chemoresistance via the E2F1-p73/DNp73-miR-205 Network. Cancer Res. 73, 3511–3524. 10.1158/0008-5472.can-12-4095 23447575

[B72] Vesely M. D. Kershaw M. H. Schreiber R. D. Smyth M. J. (2011). Natural Innate and Adaptive Immunity to Cancer. Annu. Rev. Immunol. 29, 235–271. 10.1146/annurev-immunol-031210-101324 21219185

[B73] Villaverde A. F. Pathirana D. Frohlich F. Hasenauer J. Banga J. R. (2021). A Protocol for Dynamic Model Calibration. Water Sci. Technol. 65, 1172–1178. 10.2166/wst.2012.934

[B74] Villaverde A. F. Barreiro A. Papachristodoulou A. (2016). Structural Identifiability of Dynamic Systems Biology Models. Plos Comput. Biol. 12, e1005153. 10.1371/journal.pcbi.1005153 27792726 PMC5085250

[B75] Villaverde A. F. Fröhlich F. Weindl D. Hasenauer J. Banga J. R. (2019). Benchmarking Optimization Methods for Parameter Estimation in Large Kinetic Models. Bioinformatics 35, 830–838. 10.1093/bioinformatics/bty736 30816929 PMC6394396

[B76] Yee C. Thompson J. A. Roche P. Byrd D. R. Lee P. P. Piepkorn M. (2000). Melanocyte Destruction after Antigen-Specific Immunotherapy of Melanoma. J. Exp. Med. 192, 1637–1644. 10.1084/jem.192.11.1637 11104805 PMC2193107

[B77] Zi Z. (2011). Sensitivity Analysis Approaches Applied to Systems Biology Models. IET Syst. Biol. 5, 336–346. 10.1049/iet-syb.2011.0015 22129029

