# Supplementary material for: Multi-Level Computational Modeling of Anti-Cancer Dendritic Cell Vaccination Utilized to Select Molecular Targets for Therapy Optimization
Source: Front Cell Dev Biol. 2022 Feb 2;9:746359. doi: 10.3389/fcell.2021.746359 (PMC8847669; doi:10.3389/fcell.2021.746359)
Supplement: Supplementary file 1 [file DataSheet1.pdf]

Supplementary Material

Multi-level computational modeling of anti-cancer dendritic cell vaccination utilized to select molecular targets for therapy optimization

Xin Lai[1,4,*,#], Christine Keller[1*], Guido Santos[1,2], Niels Schaft[3,4], Jan Dörrie[3,4], Julio Vera[1,4,#]

[1]Laboratory of Systems Tumor Immunology, Department of Dermatology, Friedrich-Alexander-Universität Erlangen-Nürnberg (FAU) and Universitätsklinikum Erlangen, Erlangen, Germany

[2]Departament of Biochemistry, Microbiology, Cell Biology and Genetics, Faculty of Sciences, University of La Laguna, Spain

[3]RNA Group, Department of Dermatology, Friedrich-Alexander-Universität Erlangen-Nürnberg (FAU) and Universitätsklinikum Erlangen, Erlangen, Germany

[4]Deutsches Zentrum Immuntherapie and Comprehensive Cancer Center Erlangen-EMN, Erlangen, Germany

*equal contributors

[#]Corresponding Author: xin.lai@uk-erlangen.de; julio.vera-gonzalez@uk-erlangen.de

1 Model construction

Based on the model scheme given in Figure 1 we developed a multi-level model accounting for different stages of DC-based anticancer immunotherapy. The model is composed of three parts representing different stages of the treatment – DC distribution in the human organs after injection, the biochemical pathway that regulates DC maturation, and the DC-mediated $CD8^+$ T-cell responses.

Based on previous work (Ludewig, et al. 2004) we developed a mathematical model based on ordinary differential equations (ODEs) that accounts for the trafficking and distribution of DCs in the human body. Specifically, the injected cells mainly spread into the liver, the lung, the spleen, and other peripheries (Ludewig, et al. 2004, Mackensen, et al. 1999).

Furthermore, we describe the DC maturation by the activation of the biochemical signaling pathway NF-κB. The model is also based on ODEs and adopted from our previous work (Schulz, et al. 2017). NF-κB is regulated by inhibitory proteins such as IκB. Stimulation with TNF-α, CD40L, and IL-1β leads to degradation of IκB what results in NF-κB activation. Since mature DCs secrete a variety of cytokines it was decided to concentrate on the most important ones in respect of T cell activation. IL-12 promotes IFN-γ production by T lymphocytes but it can also induce their proliferation (Henry, et al. 2008, Vecchio, et al. 2007). IFN-γ has antiviral, immunoregulatory, and anti-tumor functions (Parker, Rautela und Hertzog 2016, Tannenbaum und Hamilton 2000). The expansion and activation of T cells are also regulated by IL-6 which has a broad effect on immune cells (Hunter und Jones 2017). The chemokine IL-8 is included in the model because of its role in recruiting T helper cells (Taub, et al. 1996). DCs also express different surface molecules after stimulation. One of those is CD70. It supports the expansion of antigen-specific $CD8^+$ T cells (Arens, et al. 2004, Borst, Hendriks und Xiao 2005). The NF-κB pathway activates through the stimulation of TNFR1 and IL-1R receptors by TNF-α and IL-1β, respectively. CD40L activates the pathway through the CD40 receptor. After recognition of those proteins, recruitment of protein effectors can result in phosphorylation. For the model validation, it was necessary to add a stimulation with LPS which activates the TLR-4 receptor.

The last step of our model accounts for the DC-mediated activation of $CD8^+$ T cells in the spleen. An adult human contains approximately around $(3\text{-}5)\cdot 10^{11}$ naive T cells (Abbas, Lichtman und Pillai 2018, Jenkins, et al. 2010), whereof one cell out of 10^5-10^6 expresses a sufficient TCR for a specific antigen (Celli, et al. 2012, Henrickson, et al. 2008). After the naive T cells have matured in the

thymus they continuously move through the blood, lymphatic vessels, secondary lymphoid organs like the spleen and non-lymphoid tissue for antigen recognition. After homing into the spleen, they spend around 24 h in the T cell zones, where the interaction with DCs occurs (Celli, et al. 2012, Nolz 2015). The T cell activation happens in three phases (Henrickson, et al. 2008, Ozga, et al. 2016): the first phase is a short-term interaction between the naive T cell and the DC, it lasts about 8h and the T cell starts to upregulate activation markers. The second phase, which lasts about 12 h, is accompanied by further upregulation of activation markers and initiation of IFNγ and IL-2. The third phase begins about 24 h after the first contact, during this short T cell-DC interaction the T cell proliferation is induced. In addition, it was assumed that the activated T cells also remain in the spleen for about 24 h, as they usually migrate to the site of infection immediately after activation. In addition, the mortality rate of the naive T cells can be neglected since they have a half-life of several years (Jenkins, et al. 2010, Vrisekoop, et al. 2008) and in this study, only a period of a few weeks is considered. It is not yet fully understood whether T cells follow a linear or asymmetric differentiation pathway (Ahmed, et al. 2009). Nevertheless, this work will follow an asymmetric approach as proposed in several other works and models (Arsenio, et al. 2013, Crauste, et al. 2017, Joshi, et al. 2007, Girel, et al. 2007). Thus, DC-induced naive T cell activation leads to differentiation into early effector CD8 T cells. Those cells proliferate after the second contact with antigen-presenting cells as described in the above paragraph. After some time, the early effectors differentiate into short-lived effector cells and memory precursor cells generating long-lived memory cells. The process of whether the early effector cells become short-lived effector or memory precursor cells is supported by the available amount of inflammatory cytokines like IL-12 during priming, i.e. high inflammation induces the generation of more short-lived effector cells (Joshi, et al. 2007, Pearce und Shen 2007). Short-lived effector CD8 T cells degrade with a half-life of approximately 65-80 days (Joshi, et al. 2007), thereby around 10% become long-lived memory cells (Mueller, et al. 2013, Badovinac, Porter und Harty 2002). In addition, two different types of long-lived memory T cells can be distinguished: effector memory T cells and central memory T cells (Arsenio, et al. 2013, Wherry, et al. 2003). It is still controversial under which conditions effector memory T cells and central memory T cells occur (Ahmed, et al. 2009), thus there is no reliable data available. Therefore, memory precursors and all kinds of long-lived memory cells are generally referred to as memory T cells within this work.

In the following, the model equations are presented. For better readability, the model is divided into the three main components in the paragraphs below.

1.1 DC distribution

The following system of ordinary differential equations (ODE) describes the distribution of DCs in the human body after injection. We describe the temporal cell dynamics in the blood, the spleen, the liver, and the lung.

$$\frac{d}{dt}DC_{Blood}(t) = -\mu DC_{Blood}(t), \text{with} \mu = \mu_{BLI} + \mu_{BS} + \mu_{BLU} + \mu_{B0} \quad (1)$$

Equation (1) accounts for gradual decrease of the DCs in the blood $DC_{Blood}(t)$. Where the parameter μ describes the total distribution rate into the other compartments. μ_{BO} denotes the homing rate into other periphery.

$$\frac{d}{dt}DC_{Spleen}(t) = \mu_{BS}\frac{Q_{Blood}}{Q_{Spleen}}DC_{Blood}(t) - \mu_{S0}DC_{Spleen}(t) \quad (2)$$

Equation (2) shows the temporal evolution of DCs in the spleen $DC_{Spleen}(t)$. Thereby the cells have a homing rate of μ_{BS} from blood into the spleen and leave the compartment with a rate of μ_{S0}. The splenic volume is given by Q_{Spleen}.

$$\frac{d}{dt}DC_{Liver}(t) = \mu_{BLI}\frac{Q_{Blood}}{Q_{Liver}}DC_{Blood}(t) \quad (3)$$

The DC distribution into the liver $DC_{Liver}(t)$ is given by equation (3). The amount of cells remains constant over time (Mackensen, et al. 1999), while the homing rate from blood to liver is given by μ_{BLI}. The volume of the liver is denoted by Q_{Liver}.

$$\frac{d}{dt}DC_{Lung}(t) = \mu_{BLU}\frac{Q_{Blood}}{Q_{Lung}}DC_{Blood}(t) - \mu_{LU0}\left(DC_{Lung}(t) - DC_T\right) \quad (4)$$

Equation (4) displays the DC activity in the lung $DC_{Lung}(t)$. It was found that the cell number does not shrink below a certain threshold denoted by DC_T (Mackensen, et al. 1999). The homing rate is given by μ_{BLU} whereby the lung is cleared again with a rate of μ_{LU0} and Q_{Lung} is the volume of the lung. The initial concentrations of DC in the spleen, lung and liver were assumed to be zero.

All four ordinary differential equations can be solved analytically by separation of variables or variation of constants if the equation is nonlinear. Thereby the following system of equations is obtained:

$$DC_{Blood}(t) = \frac{DC_{in}}{Q_{Blood}}e^{-\mu t} \quad (5)$$

$$DC_{Spleen}(t) = \frac{\mu_{BS} DC_{in}}{Q_{Spleen}} \frac{1}{(\mu - \mu_{S0})} (e^{-\mu_{S0} t} - e^{-\mu t}) \tag{6}$$

$$DC_{Liver}(t) = \frac{\mu_{BLI} DC_{in}}{Q_{Liver}} \frac{1}{\mu} (1 - e^{-\mu t}) \tag{7}$$

$$\begin{aligned} DC_{Lung}(t) = DC_T & \\ &+ e^{-\mu_{LU0} t} \left[\frac{\mu_{BLU} DC_{in}}{Q_{Lung}} \frac{1}{-(\mu - \mu_{LU0})} e^{-(\mu - \mu_{LU0}) t} \right. \\ &\left. + \frac{\mu_{BLU} DC_{in}}{Q_{Lung}} \frac{1}{(\mu - \mu_{LU0})} - DC_T \right] \end{aligned} \tag{8}$$

With the initial conditions chosen as follows:

$$DC_{Blood}(t = 0) = \frac{DC_{in}}{Q_{Blood}} \tag{9}$$

$$DC_{Spleen}(t = 0) = 0 \tag{10}$$

$$DC_{Liver}(t = 0) = 0 \tag{11}$$

$$DC_{Lung}(t = 0) = 0 \tag{12}$$

1.2 DC activation

After stimulation with TNF-α, IL-1β, CD40L, or LPS the respective receptor-associated proteins are phosphorylated which leads to activation of IKK. Thereby IκBα is degraded which results in the release of free NF-κB. The transcription factor NF-κB enters the nucleus where the transcription of many genes is upregulated. Among these genes, the model accounts for IκBα and the most important ones for T-cell activation. This further leads to the secretion of chemokines and cytokines, and the upregulation of surface molecules, i.e., IL-12p70 mRNA, IL-6 mRNA, IL-8 mRNA, and CD70 mRNA.

The phosphorylation of IRAK1 and TRAF2 is described as follows:

$$\begin{aligned} \frac{\mathrm{d}}{\mathrm{dt}} \mathrm{IRAK1(t)} = k_{syn}^{IRAK1} - k_{deg}^{IRAK1} \cdot \mathrm{IRAK1(t)} - k_{ph1}^{IRAK1} \cdot \mathrm{IRAK1(t)} \cdot \mathrm{IL1}\beta \\ - k_{ph2}^{IRAK1} \cdot \mathrm{IRAK1(t)} \cdot \mathrm{LPS} \end{aligned} \tag{13}$$

$$\begin{aligned} \frac{\mathrm{d}}{\mathrm{dt}} \mathrm{IRAK1p(t)} = k_{ph1}^{IRAK1} \cdot \mathrm{IRAK1}(t) \cdot \mathrm{IL1}\beta + k_{ph2}^{IRAK1} \cdot \mathrm{IRAK1}(t) \cdot \mathrm{LPS} \\ - k_{deg}^{IRAK1p} \cdot \mathrm{IRAK1p}(t) \end{aligned} \tag{14}$$

$$\frac{\mathrm{d}}{\mathrm{dt}} \mathrm{TRAF2(t)} = k_{syn}^{TRAF2} - k_{deg}^{TRAF2} \cdot \mathrm{TRAF2}(t) - k_{ph1}^{TRAF2} \cdot \mathrm{TRAF2}(t) \cdot \mathrm{TNF}\alpha \tag{15}$$

$$- k_{ph2}^{TRAF2} \cdot \mathrm{TRAF2}(t) \cdot \mathrm{CD40L}$$

$$\frac{\mathrm{d}}{\mathrm{dt}}\mathrm{TRAF2p(t)} = k_{ph1}^{TRAF2} \cdot \mathrm{TRAF2}(t) \cdot \mathrm{TNF}\alpha + k_{ph2}^{TRAF2} \cdot \mathrm{TRAF2}(t) \cdot \mathrm{CD40L} - k_{deg}^{TRAF2p} \cdot \mathrm{TRAF2p}(t) \quad (16)$$

Stimulation with IL1-β and LPS lead to phosphorylation of IRAK1 with the rates k_{ph1}^{IRAK1} and k_{ph2}^{IRAK1}, respectively. The synthesis and degradation rate of IRAK1 are denoted by k_{syn}^{IRAK1} and k_{deg}^{IRAK1}, respectively. IRAK1p describes the phosphorylated state with a degradation rate of k_{deg}^{IRAK1p}.

This is equivalent for the phosphorylation of TRAF2 by the stimulation with TNF-α and CD40L. Here the phosphorylated state is denoted with TRAF2p.

This process leads to a state transition of IKK:

$$\frac{\mathrm{d}}{\mathrm{dt}}\mathrm{IKK(t)} = k_{syn}^{IKK} - k_{act1}^{IKK} \cdot \mathrm{IKK}(t) \cdot \mathrm{IRAK1p}(t) - k_{act2}^{IKK} \cdot \mathrm{IKK}(t) \cdot \mathrm{TRAF2p}(t) - k_{deg}^{IKK} \cdot \mathrm{IKK}(t) \quad (17)$$

Where k_{syn}^{IKK} is the synthesis rate of IKK, $k_{act1}^{IKK}$and k_{act2}^{IKK} are the respective activation rates of IKK through the phosphorylated proteins IRAK1p and TRAF2p. The degradation rate is given by k_{deg}^{IKK}.

Activation of IKK results in IKKβ increase, which is degraded with a rate k_{deg}^{IKKb}:

$$\frac{\mathrm{d}}{\mathrm{dt}}\mathrm{IKK}\beta\mathrm{(t)} = k_{act}^{IKK} \cdot \mathrm{IKK}(t) \cdot \mathrm{IRAK1p}(t) + k_{act}^{IKK} \cdot \mathrm{IKK}(t) \cdot \mathrm{TRAF2p}(t) - k_{deg}^{IKKb} \cdot \mathrm{IKK}\beta(t) \quad (18)$$

This activation can release free NF-κB by degrading IκBα:

$$\frac{\mathrm{d}}{\mathrm{dt}}\mathrm{NF}\kappa\mathrm{B(t)} = k_{gain}^{NF\kappa B} \cdot IKK\beta(t) \cdot \frac{N_{tot} - \mathrm{NF}\kappa\mathrm{B}(t)}{K_1 + \mathrm{I}\kappa\mathrm{B}\alpha(t) + \mathrm{IKK}\beta(t)} - k_{loss}^{NF\kappa B} \cdot \frac{\mathrm{I}\kappa\mathrm{B}\alpha(t) \cdot \mathrm{NF}\kappa\mathrm{B}(t)}{K_2 + \mathrm{NF}\kappa\mathrm{B}(t)} \quad (19)$$

$k_{gain}^{NF\kappa B}$ is the gain rate of free NF-κB in the nucleus mediated by IκBα and IKKβ and $k_{loss}^{NF\kappa B}$ is the loss of free NF-κB mediated by IκBα. The total amount of free NF-κB is given by N_{tot}. K_1 and K_2 are the Michaelis-Menten like coefficients.

The transcription factor NF-κB enters the nucleus and upregulates the transcription of many genes, including the transcription of gene for IκBα into mRNA (mIκBα):

$$\frac{\mathrm{d}}{\mathrm{dt}}\mathrm{mI}\kappa\mathrm{B}\alpha\mathrm{(t)} = k_{transc}^{mI\kappa B\alpha} \cdot \mathrm{NF}\kappa\mathrm{B}(t) - k_{deg}^{mI\kappa B\alpha} \cdot \mathrm{mI}\kappa\mathrm{B}\alpha(t) \quad (20)$$

With transcription rate $k_{transc}^{mI\kappa B\alpha}$ and degradation rate $k_{deg}^{mI\kappa B\alpha}$.

This mRNA is further translated into IκBα which again leads to down regulation of NF-κB:

$$\frac{\mathrm{d}}{\mathrm{dt}}\mathrm{I}\kappa\mathrm{B}\alpha(\mathrm{t}) = k_{transl}^{I\kappa B\alpha} \cdot \mathrm{mI}\kappa\mathrm{B}\alpha(t) - k_{loss}^{I\kappa B\alpha} \cdot \mathrm{IKK}\beta(t) \cdot \mathrm{I}\kappa\mathrm{B}\alpha(t) \cdot \frac{N_{tot} - \mathrm{NF}\kappa\mathrm{B}(t)}{K_3 + \mathrm{I}\kappa\mathrm{B}\alpha(t) + \mathrm{IKK}\beta(t)} \tag{21}$$

$k_{transl}^{I\kappa B\alpha}$ denotes the translation of mIκBα into IκBα whereby the $k_{loss}^{I\kappa B\alpha}$is the loss of free IκBα mediated by NF-κB and IKKβ. K_3 is the Michaelis-Menten like coefficient.

The transcription of mIL-8 and mIL-6 is also upregulated:

$$\frac{\mathrm{d}}{\mathrm{dt}}\mathrm{mIL8}(\mathrm{t}) = k_{transc1}^{mIL8} + k_{trancs2}^{mIL8} \cdot \mathrm{NF}\kappa\mathrm{B}(t) - k_{deg}^{mIL8} \cdot \mathrm{mIL8}(t) \tag{22}$$

$$\frac{\mathrm{d}}{\mathrm{dt}}\mathrm{mIL6}(\mathrm{t}) = k_{transc1}^{mIL6} + k_{trancs2}^{mIL6} \cdot \mathrm{NF}\kappa\mathrm{B}(t) - k_{deg}^{mIL6} \cdot \mathrm{mIL6}(t) \tag{23}$$

Whereby $k_{transc1}^{mIL8}$ and $k_{transc1}^{mIL6}$ are the basal transcription rates, $k_{transc2}^{mIL8}$ and $k_{transc2}^{mIL6}$ are the respective transcription rates induced by NF-κB and the degradation rates of mIL-8 and mIL-6 are given by $k_{deg}^{mIL8}$and k_{deg}^{mIL6}.

Those are translated into the chemokine IL-8 and the pro-inflammatory cytokines IL-6, respectively:

$$\frac{\mathrm{d}}{\mathrm{dt}}\mathrm{IL8}_{\mathrm{DC}}(t) = k_{transl}^{IL8} \cdot \mathrm{mIL8}(t) - k_{deg}^{IL8} \cdot \mathrm{IL8}_{\mathrm{DC}}(t) - k_{sec}^{IL8} \cdot \mathrm{IL8}_{\mathrm{DC}}(t) \tag{24}$$

$$\frac{\mathrm{d}}{\mathrm{dt}}\mathrm{IL6}_{\mathrm{DC}}(t) = k_{transl}^{IL6} \cdot \mathrm{mIL6}(t) - k_{deg}^{IL6} \cdot \mathrm{IL6}_{\mathrm{DC}}(t) - k_{sec}^{IL6} \cdot \mathrm{IL6}_{\mathrm{DC}}(t) \tag{25}$$

With the translation rates k_{transl}^{IL8} and k_{transl}^{IL6} and the degradation rates k_{deg}^{IL8} and k_{deg}^{IL6}. The secretion of the proteins by the DCs is mediated by a rate of k_{sec}^{IL8} for IL-8 and k_{sec}^{IL6} for IL-6.

NF-κB also stimulates mCD70 which results in a presentation of CD70 molecule on the cell surface:

$$\frac{\mathrm{d}}{\mathrm{dt}}\mathrm{mCD70}(\mathrm{t}) = k_{transc1}^{mCD70} + k_{trancs2}^{mCD70} \cdot \mathrm{NF}\kappa\mathrm{B}(t) - k_{deg}^{mCD70} \cdot \mathrm{mCD70}(t) \tag{26}$$

$$\frac{\mathrm{d}}{\mathrm{dt}}\mathrm{CD70}(\mathrm{t}) = k_{transl}^{CD70} \cdot \mathrm{mCD70}(t) - k_{deg}^{CD70} \cdot \mathrm{CD70}(t) \tag{27}$$

Again $k_{transc1}^{mCD70}$ describes a basal transcription rate, whereby $k_{transc2}^{mCD70}$ is the rate of transcription of mCD70 mediated by NF-κB.

k_{transl}^{CD70} is the translation of mCD70 into CD70 and k_{deg}^{CD70} is the degradation of the surface marker.

The cytokine IL-12p70 is not directly activated because it is a heterodimer consisting of IL-12p40 and IL-12p35 joined by bond. IL-12p40 is constantly active but IL-12p35 is activated through NF-κB. For simplicity it is estimated that the amount of IL-12p40 is very small compared to IL-12p35. So its activation can be written as a direct result in IL-12p70 translation:

$$\frac{\mathrm{d}}{\mathrm{dt}}\mathrm{mIL12p35(t)} = k_{transc1}^{mIL12p35} + k_{trancs2}^{mIL12p35} \cdot \mathrm{NF}\kappa\mathrm{B}(t) - k_{deg}^{mIL12p35} \cdot \mathrm{mIL12p35}(t) \qquad (28)$$

$$\frac{\mathrm{d}}{\mathrm{dt}}\mathrm{IL12p70_{DC}}(t) = k_{transl}^{IL12p35} \cdot \mathrm{mIL12p35}(t) - k_{deg}^{IL12p70} \cdot \mathrm{IL12p70_{DC}}(t) - k_{sec}^{IL12p70} \cdot \mathrm{IL12p70_{DC}}(t) \qquad (29)$$

With basal transcription rate $k_{transc1}^{mIL12p35}$, transcription rate for NF-κB mediated transcription $k_{transc2}^{mIL12p35}$ and degradation rate $k_{deg}^{mIL12p35}$ of mIL-12p35. As well as the translation rate of mIL-12p35 into IL-12p70 $k_{transl}^{IL12p35}$, the degredation rate $k_{deg}^{IL12p70}$ and the secretion rate $k_{sec}^{IL12p70}$ of IL-12p70.

The secretion of the cytokines can be described as follows:

$$\frac{\mathrm{d}}{\mathrm{dt}}\mathrm{IL12p70(t)} = k_{sec}^{IL12p70} \cdot \mathrm{IL12p70_{DC}}(t) - k_{biodist}^{IL12p70} \cdot \mathrm{IL12p70}(t) \qquad (30)$$

$$\frac{\mathrm{d}}{\mathrm{dt}}\mathrm{IL6(t)} = k_{sec}^{IL6} \cdot \mathrm{IL6_{DC}}(t) - k_{biodist}^{IL6} \cdot \mathrm{IL12p70}(t) \qquad (31)$$

$$\frac{\mathrm{d}}{\mathrm{dt}}\mathrm{IL8(t)} = k_{sec}^{IL8} \cdot \mathrm{IL8_{DC}}(t) - k_{biodist}^{IL8} \cdot \mathrm{IL8}(t) \qquad (32)$$

Where $k_{sec}^{IL12p70}, k_{sec}^{IL6}$ and k_{sec}^{IL8} are the distribution rates for IL-12p70, IL-6, and IL-8 in blood, respectively.

The initial amount of the variables was chosen to be zero, except for IRAK1(t = 0), TRAF2(t = 0), IKK(t = 0), and IκBα(t = 0). Those initial values were set equal to one. If activation through Lipopolysaccharide (LPS) is necessary the respective parameter has to be set equal to one, whereof the other input signals TNFα, CD40L, and IL1-β have to be zero. For activation through TNFα, CD40L, and IL1-β these values have to be chosen as one and LPS equal to zero.

1.3 DC-mediated T-cell response

The last part of the model describes the dynamics of T cell activation through contact with DCs. Thereby we concentrate on the spleen, as we currently have no data available for other lymphoid organs. Entering the spleen DCs travel to T-cell zones where the interaction takes place. After successful contact between DCs and naive T cells, the T cells are activated and differentiate into early effector T cells. After second contact, early effector cells proliferate and further differentiate into short-lived effector cells and long-lived memory cells. Whereof short-lived effector cells have a half-life of approximately 65-80 days and around 10 % become memory T cells (Mueller, et al. 2013, Badovinac, Porter und Harty 2002). We will neglect the homing of activated short-lived effector and

memory T cells into other compartments or the tumor microenvironment since we are mainly interested in the total amount of memory cells.

All together results in the following system of differential equations accounting for the DC-T cell interaction:

$$\frac{\mathrm{d}}{\mathrm{dt}}N(t) = k_{hom}^{N} \cdot N_0 - k_{act}^{N} \cdot N(t) \cdot \frac{DC_{spleen}(t) \cdot F(t)}{K_4 + DC_{Spleen}(t) \cdot F(t)} - k_{dist}^{N} \cdot N(t) \tag{33}$$

$$\frac{\mathrm{d}}{\mathrm{dt}}EE(t) = k_{act}^{N} \cdot N(t) \cdot \frac{DC_{Spleen}(t) \cdot F(t)}{K_4 + DC_{spleen}(t) \cdot F(t)} + k_{prol}^{EE} \cdot EE(t-\tau) \cdot \frac{DC_{Spleen}(t) \cdot F(t)}{K_4 + DC_{spleen}(t) \cdot F(t)} - k_{diff1}^{EE} \cdot \mathrm{IL12p70}(t) \cdot EE(t) - k_{diff2}^{EE} \cdot EE(t) \tag{34}$$

$$\frac{\mathrm{d}}{\mathrm{dt}}SLE(t) = k_{diff1}^{EE} \cdot \mathrm{IL12p70}(t) \cdot EE(t) - k_{deg}^{SLE} \cdot SLE(t) \tag{35}$$

$$\frac{\mathrm{d}}{\mathrm{dt}}M(t) = k_{diff2}^{EE} \cdot EE(t) + 0.1 \cdot k_{deg}^{SLE} \cdot SLE(t) \tag{36}$$

Equation (**33**) describes the activation of naive T cells (N). Thereby, the cells home into the spleen with a rate of k_{hom}^{N}. N_0 is the initial value for naive cells that get activated with a rate of k_{act}^{N}. The activation depends on the amount of available DCs in the spleen (DC_{spleen}) and the intensity of cytokines, chemokines, and surface markers produced or presented by DCs characterized by the following function

$$F(t) = \mathrm{IL12p70}(t) \cdot \mathrm{IL6}(t) \cdot \mathrm{IL8}(t) \cdot \mathrm{CD70}(t) \tag{37}$$

Equation (37) is the product of IL-12p70, IL-6, IL-8, and CD70. The naive T cells leave the spleen with a distribution rate of k_{dist}^{N}. K_4 is the Michaelis-Menten like constant.

Equation (**34**) describes the dynamics of early effector T cells (EE). Whereof the early effector cell proliferation is expressed by a delay differential equation $EE(t-\tau)$ with a rate of k_{prol}^{EE} and a delay of τ. Early effector T cells can differentiate into short-lived effector cells (SLE) with a rate of k_{diff1}^{EE} and the process is dependent on IL-12p70. Besides, early effector T cells can differentiate into memory T cells (M) with a rate of k_{diff2}^{EE}.

The temporal dynamics of SLE is characterized by equation (**35**), where k_{deg}^{SLE} is the degradation rate. A fraction (10%) of degradingSLE becomes memory T cells.

Equation (**36**) describes the generation of memory T cells (M) that are transformed from early effector T cells (EE) and short-lived effector cells (SLE).

The initial conditions of the model variables are given as follows:

$$N(t=0)=N_0 \quad (38)$$

$$EE(t=0)=0 \quad (39)$$

$$SLE(t=0)=0 \quad (40)$$

$$M(t=0)=0 \quad (41)$$

2 Parameter estimation for different parts of the model

To calibrate the model, we used experimental data to adjust the parameter values. The optimization strategy is described in the Materials and Methods. For better readability, this section is divided into three parts.

2.1 DC distribution

For calibration, the model simulation was fitted to experimental data from Mackensen et al. (Mackensen, et al. 1999). The data describes the distribution of *in vitro* differentiated DCs into the liver, the spleen, the lung and other periphery after intravenous injection. The result is given in Figure 2.

2.2 DC activation

Since it was not possible to find enough data to fit the model some parameters were kept constant to reduce their number and to prevent overfitting. Synthesis rates were set equal to the degradation rates to make the steady states equal to the initial values when no input to the system is given. Further, the translation constants $k_{trans1}^{mIL12p70}, k_{trans1}^{mIL6}$ and k_{trans1}^{mIL8} could be set to zero because the proteins are not basal as shown by Pfeiffer et al. (Pfeiffer, et al. 2014). The values for K_1, K_2 and K_3 were set equal to one for simplicity. Since so far, no dynamical data for the NF-κB pathway activation in dendritic cells with activation through TNF-α, CD40L, or IL-1β was found, but with LPS. Therefore, we used the experimental data from Bode et al. (Bode, Schmitz, et al. 2008) to calibrate the model and used LPS as only the input signal. The data contain information about the dynamics of IκBα mRNA, IκBα, and NF-κB. For the IκBα fitting, additional simulation points were added since the given data did not account for the entire period being simulated. The result for the model simulation compared to the data is given in Figure 3. To validate the dynamics for the cytokines IL-12p10 and IL-6, the chemokine IL-8, and the surface marker CD70 we used the data set from Pfeiffer et al. (Pfeiffer, et al. 2014). Therefore, TNF-α, CD40L, and IL-1β but no LPS were used as input signals. The outcome is

shown in Figure 4. The concentrations were measured 24 h after electroporation with caIKKβ-RNA. To simulate electroporated DCs we introduced a modified degradation rate of IKKβ, i.e., $k_{deg,mod}^{IKKb}$. We also adjusted this parameter value to calibrate the model.

2.3 DC-mediated T-cell responses

To calibrate the DC- T cell interaction part of the model experimental data from Pfeiffer et al. (Pfeiffer, et al. 2014) is used. In this work $1 \cdot 10^6$ CD8⁺ T cells are primed in vitro with Mock-electroporated and caIKKα- and caIKKβ-mRNA (alone or in combination) electroporated DCs. The DCs were used four hours after electroporation to stimulate the T cells at a ratio 1:10. After one week the total number of MelA-specific $CD8^+$ T cells was determined. The T cells were restimulated two times. Those caIKKα- and caIKKβ-mRNA electroporated DCs have up to 10 times higher cytokine/chemokine production and can stimulate nearly five times more T cells (Pfeiffer, et al. 2014).

For the model calibration caIKKβ-RNA electroporated DCs are simulated by the modified degradation rate of IKKβ, i.e., $k_{deg,mod}^{IKKb}$. To simulate the experimental conditions in the model, the above-introduced system of equations is reduced to:

$$\frac{\mathrm{d}}{\mathrm{dt}}N(t) = -k_{act}^{N} \cdot N \cdot \frac{DC_{init} \cdot F(t)}{K_4 + DC_{init} \cdot F(t)} \tag{42}$$

$$\frac{\mathrm{d}}{\mathrm{dt}}EE(t) = k_{act}^{N} \cdot N \cdot \frac{DC_{init} \cdot F(t)}{K_4 + DC_{init} \cdot F(t)} + k_{prol}^{EE} \cdot EE(t-\tau) \cdot \frac{DC_{init} \cdot F(t)}{K_4 + DC_{init} \cdot F(t)} \tag{43}$$

With the number of DCs fixed to $DC_{init} = 1 \cdot 10^5$. To fit the reduced model to the data, the parameter K_4 was optimized. The results of the model calibration is given in Figure 5. Both model variations were optimized in parallel.

Sensitivity Analysis

We performed global sensitivity analyses to quantify the impact of model parameters on the dynamics of the system. We used the Sobol method that considers variations within the entire variability space of the model parameters (Saltelli, et al. 2008, Sarrazin, Pianosi und Wagener 2016). The analysis was performed using the MATLAB toolbox *SAFE* (Pianosi, Beven, et al. 2016).

Specifically, the contribution to the variance of model output (e.g., a variable accounting for the number of immune cells) by an individual input factor (i.e., a model parameter denoting the degradation of a protein) was considered a good measure for sensitivity in our analysis. Considering the following definition for the model response

$$y = g(\vec{x}) = g(x_1, \dots, x_M) \tag{44}$$

with y the model output, $\vec{x} = (x_1, \dots x_M)$ the vector containing the input factors, M the number of input factors, and g a function mapping input to output, there are different ways to calculate this contribution: first-order indices (main-effects) and total-order indices (total-effects) are considered.

By definition (Saltelli, et al. 2008, Sarrazin, Pianosi und Wagener 2016), the first-order indices are calculated using the following equation

$$S_i^F = \frac{V_{x_i}\left(E_{x_{\sim i}}(y|x_i)\right)}{V(y)} = \frac{V(y)}{V(y)-E_{x_i}}, \tag{45}$$

and the total-indices are defined as follows

$$S_i^T = \frac{E_{x_{\sim i}}\left(V_{x_i}(y|x_{\sim i})\right)}{V(y)} = 1 - \frac{V_{x_{\sim i}}\left(E_{x_i}(y|x_{\sim i})\right)}{V(y)}. \tag{46}$$

Where x_i is the i-th input factor and $x_{\sim i}$ is the vector of all input factors except the i-th one. $E(\cdot)$is the expectation value and $V(\cdot)$ is the variance of the corresponding variable. The main effect measures the direct contribution from an individual input factor to the output variance, while the total effect measures the overall contribution including the direct effect and the amplification of this effect due to interaction with all other input factors (Sarrazin, Pianosi und Wagener 2016). We applied the following workflow to perform the sensitivity analysis for the model (Pianosi, Sarrazin und Wagener, A Matlab toolbox for Global Sensitivity Analysis 2015) :

1. Two independent input samples x_A, x_B are generated. For example, x can be the parameter denoting the degradation rate of IKKβ, i.e., $x = k_{deg}^{IKK}$.
2. A matrix $x_C$is build by recombining x_A and x_B.
3. The model outputs y_A, y_B and y_C are generated. For example, y can be the total number of memory cells, such that $y = \int_{t_0}^{t_{end}} M(t)dt$.
4. First-order and total-order indices are calculated.

We generated the input samples using the Latin-Hypercube sampling method that not only samples random parameter values but also guarantees a uniform distribution of parameter values in their defined ranges (Sarrazin, Pianosi und Wagener 2016). Assuming a random sampling of size n thereby leads to

$N = n(M + 2)$ model evaluations due to the resampling strategy (Sarrazin, Pianosi und Wagener 2016). We chose n to be 2500.

To assess the uncertainty of the global sensitivity analysis, we investigated the convergence of the sensitivity indices using the *SAFE* toolbox. A value of the width of the confidence interval (here 95%) close to zero indicates that the sensitivity index converges (Pianosi, Beven, et al. 2016). The maximum width of the confidence interval was calculated by

$$Stat_{indices} = max_{i=1 \dots M}\left(S_i^{ub} - S_i^{lb}\right) \quad (47)$$

where S_i^{ub} and S_i^{lb} are the upper and lower bounds of the sensitivity index of the i-th input factor.

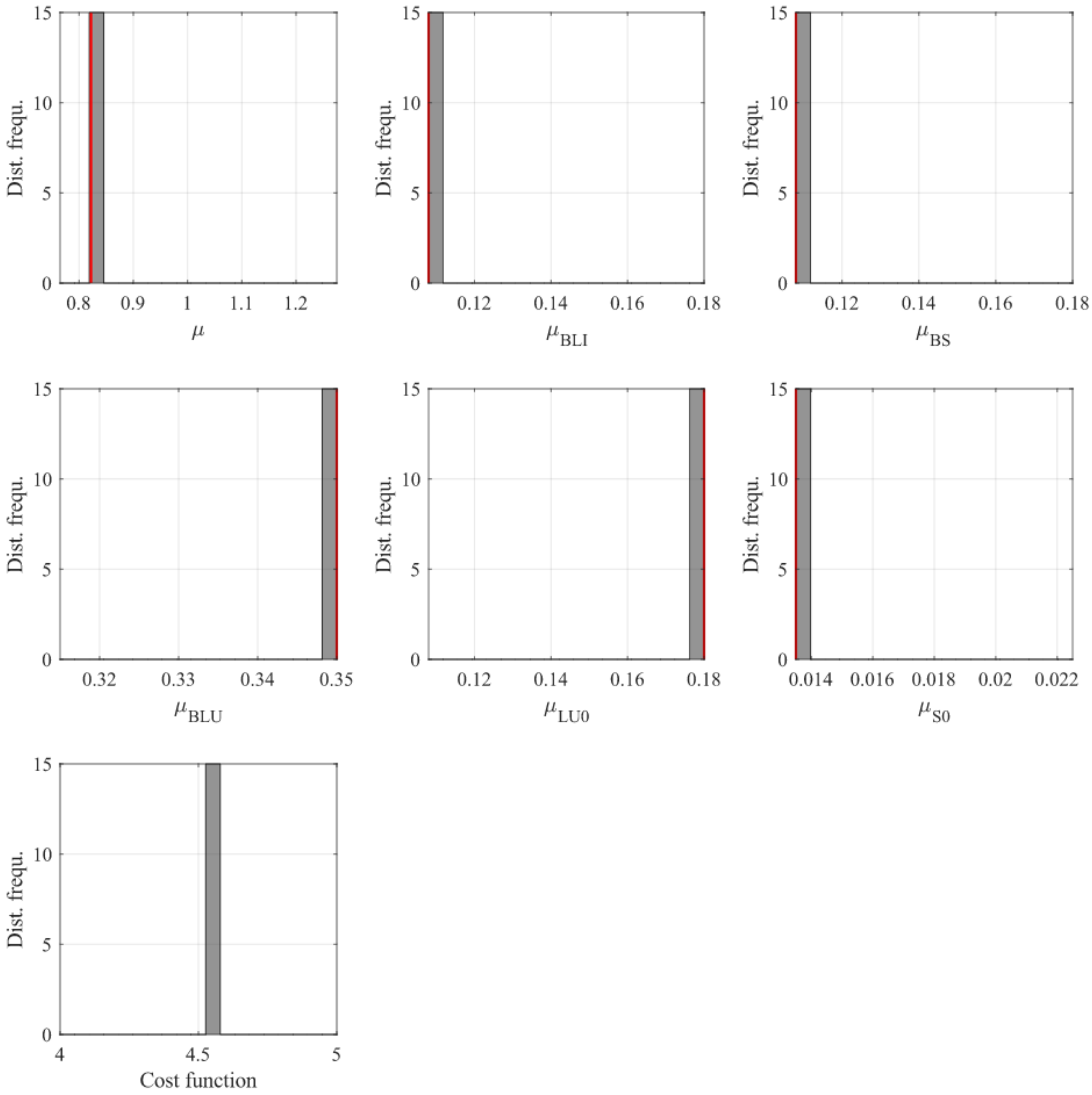

Figure S1. Parameter estimates for DC distribution in human organs. The histograms show the distribution of the estimated parameter values using the best 15 estimates. The red lines show the optimal values of estimated parameters.

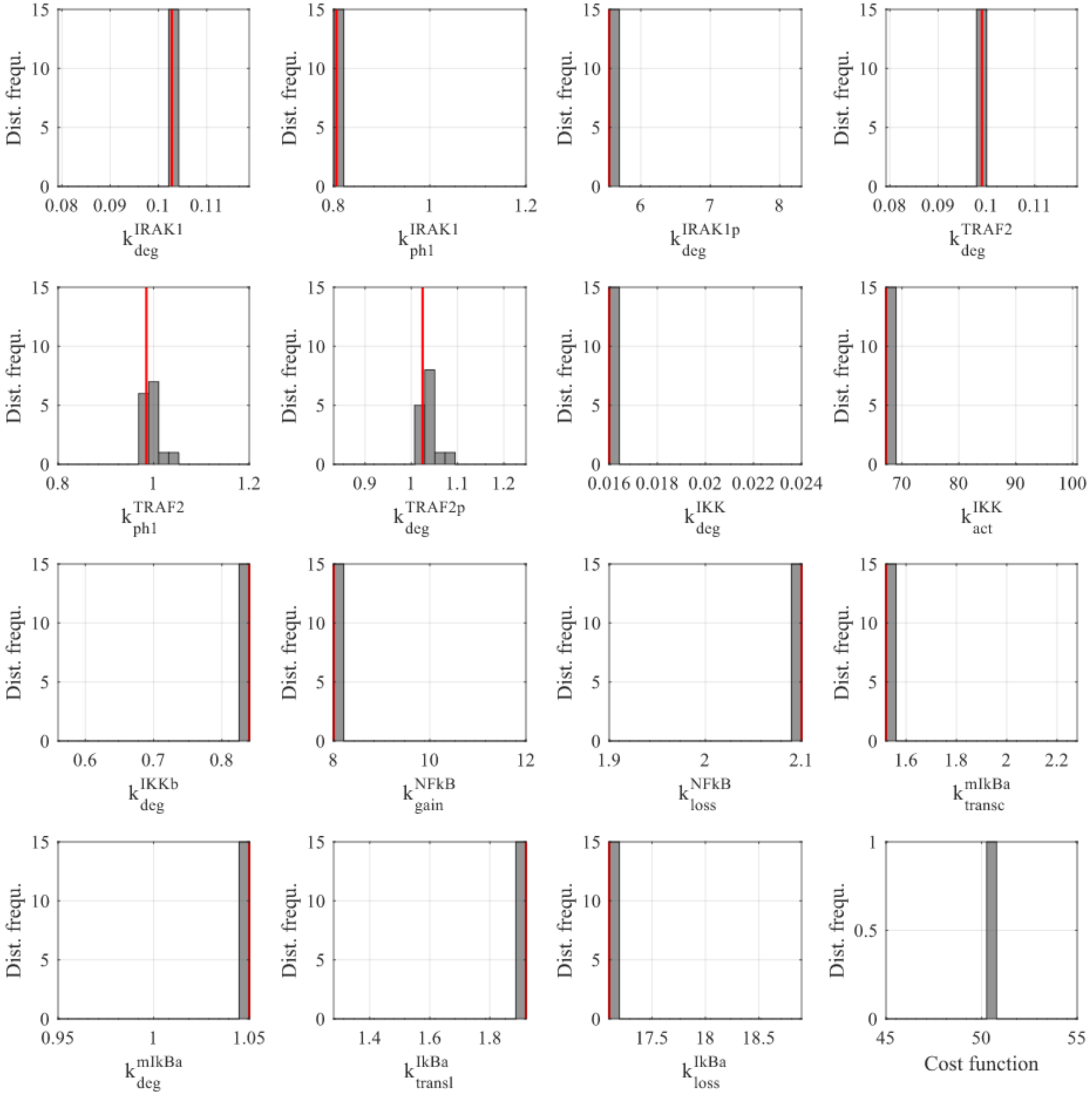
Dist. frequ.
k_{deg}^{IRAK1}
k_{ph1}^{IRAK1}
k_{deg}^{IRAK1p}
k_{deg}^{TRAF2}
k_{ph1}^{TRAF2}
k_{deg}^{TRAF2p}
k_{deg}^{IKK}
k_{act}^{IKK}
k_{deg}^{IKKb}
k_{gain}^{NFkB}
k_{loss}^{NFkB}
k_{transc}^{mIkBa}
k_{deg}^{mIkBa}
k_{transl}^{IkBa}
k_{loss}^{IkBa}
Cost function

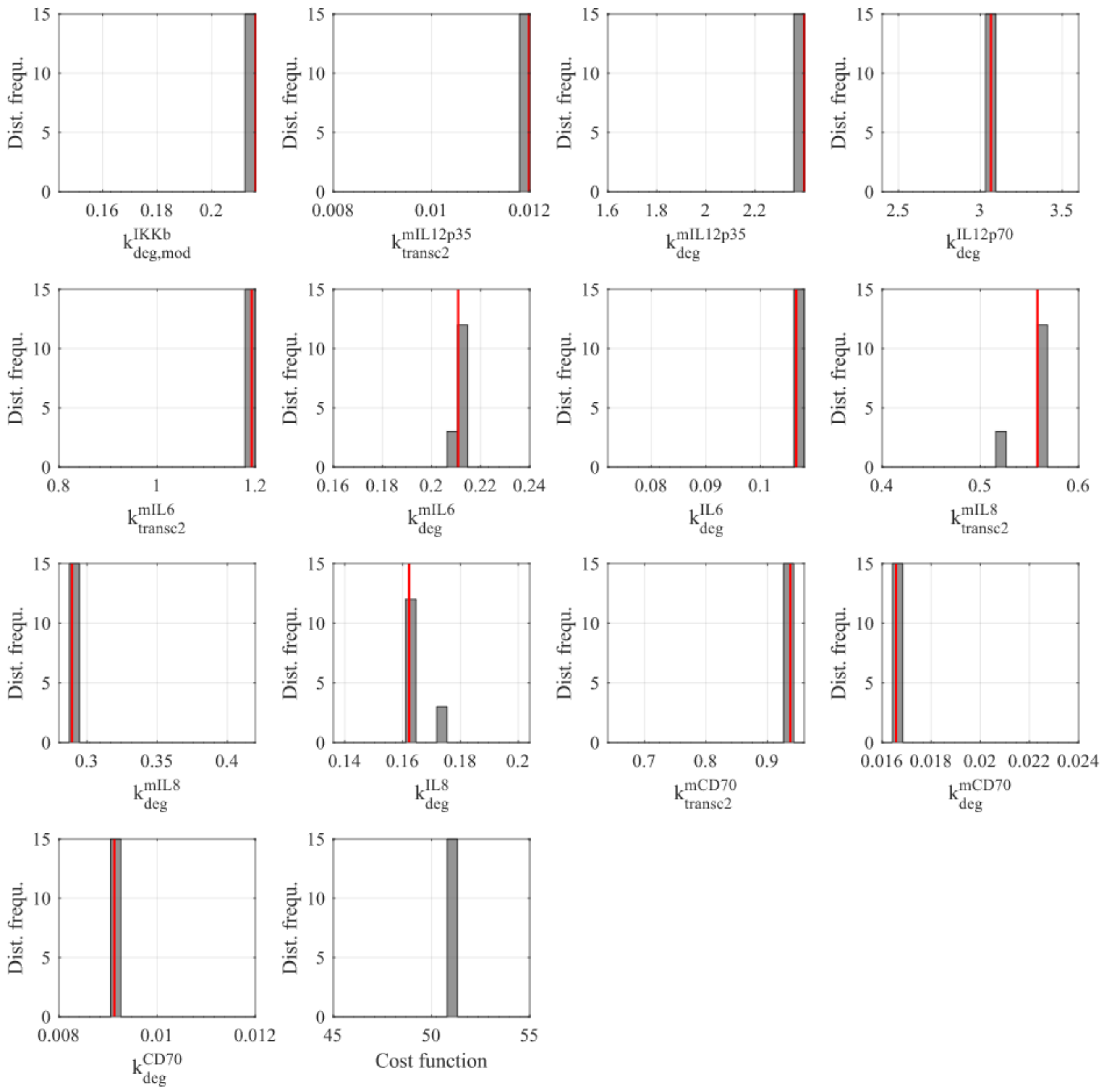

Figure S2. Parameter estimates for the NF-κB activation pathway. The histograms show the distribution of the estimated parameter values using the best 15 estimates. The red lines show the optimal values of estimated parameters.

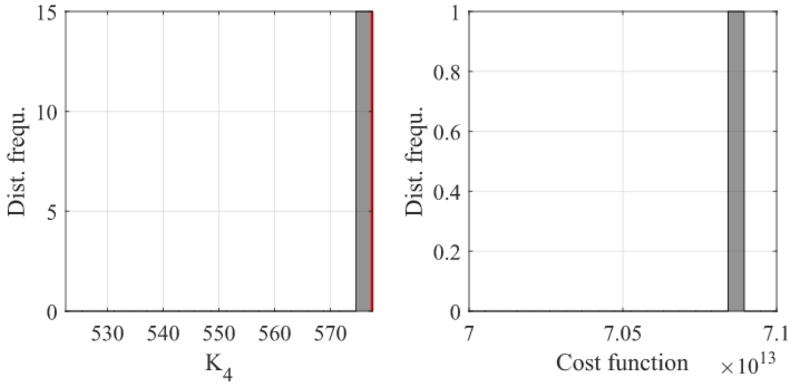

Figure S3. Parameter estimates for DC-mediated T cell response. The histograms show the distribution of the estimated parameter values using the best 15 estimates. The red lines show the optimal values of estimated parameters.

Table S1. Model parameters for the dendritic cell distribution.

Parameter	Description	Value	Range	95% CI	References	Notes
$\boldsymbol{\mu}$	DC emigration rate from blood into other compartments	0.822 h^{-1}	fitted: (0.765 – 1.275) h^{-1}	[0.822 0.822]	-	Human experiment
$\boldsymbol{\mu_{BLI}}$	homing rate into liver	0.108 h^{-1}	half-life 6h: (0.108 – 0.180) h^{-1}	[0.108 0.108]	(Mackensen, et al. 1999)	Human experiment
$\boldsymbol{\mu_{BS}}$	homing rate into spleen	0.108 h^{-1}	half-life 6h: (0.108 – 0.180) h^{-1}	[0.108 0.108]	(Mackensen, et al. 1999)	Human experiment
$\boldsymbol{\mu_{BLU}}$	homing rate into lung	0.35 h^{-1}	half-life 2h: (0.315 – 0.35) h^{-1}	[0.35 0.35]	(Mackensen, et al. 1999)	Human experiment
$\boldsymbol{\mu_{LU0}}$	DC decay in the lung	0.180 h^{-1}	half-life 6h: (0.108 – 0.180) h^{-1}	[0.180 0.180]	(Mackensen, et al. 1999)	Human experiment
$\boldsymbol{\mu_{S0}}$	DC decay in the spleen	0.0135 h^{-1}	half-life 70: (0.0135 – 0.0225) h^{-1}	[0.0135 0.0135]	(Mackensen, et al. 1999)	Human experiment
$\boldsymbol{Q_{Liver}}$	volume liver	$0.2 \cdot Q_{Blood}$	fixed	-	(Sauermost und Freudig 1999)	Human data
$\boldsymbol{Q_{Spleen}}$	volume spleen	$0.16 \cdot Q_{Blood}$	fixed	-	(Sauermost und Freudig 1999)	Human data
$\boldsymbol{Q_{Lung}}$	volume lung	$0.3 \cdot Q_{Blood}$	fixed	-	(Sauermost und Freudig 1999)	Human data
$\boldsymbol{Q_{Blood}}$	volume blood	6-7 liter	-	-	(Sauermost und Freudig 1999)	Human data
$\boldsymbol{DC_{in}}$	Number of DCs injected in blood	$1 \cdot 10^7$cells	fixed	-	(Mackensen, et al. 1999)	Human data
$\boldsymbol{DC_T}$	number of DCs that stay in lung	$0.13 \cdot 10^7$cells	$0.13 \cdot DC_{in}$	-	(Mackensen, et al. 1999)	Human data

Table S2. Model parameters for the activation of dendritic cells.

Parameter	Description	Value	Range	95% CI	References	Notes
$\boldsymbol{k_{syn}^{IRAK1}}$	synthesis rate of IRAK1	k_{ph1}^{IRAK1}	-	-	-	
$\boldsymbol{k_{deg}^{IRAK1}}$	Degradation rate of IRAK1	0.103 h^{-1}	half-life 7 h: (0.0792 – 0.1188) h^{-1}	[0.103 0.103]	(Schulz, et al. 2017)	Human cell line
$\boldsymbol{k_{ph1}^{IRAK1}}$	IL-1β mediated phosphorylation rate of IRAK1	0.81 h^{-1}	fitted: (0.80 – 1.20) h^{-1}	[0.81 0.81]	-	
$\boldsymbol{k_{ph2}^{IRAK1}}$	LPS mediated phosphorylation rate of IRAK1	k_{ph1}^{IRAK1}	-	-	-	
$\boldsymbol{k_{deg}^{IRAK1p}}$	degradation rate of phosphorylated IRAK1	5.54 h^{-1}	half-life 6 min: (5.54 – 8.32) h^{-1}	[5.54 5.54]	(Schulz, et al. 2017)	Human cell line
$\boldsymbol{k_{syn}^{TRAF2}}$	Synthesis rate of TRAF2	k_{deg}^{TRAF2}	-	-	-	
$\boldsymbol{k_{deg}^{TRAF2}}$	Degradation rate of TRAF2	0.099 h^{-1}	half-life 7h: (0.0792 – 0.1188) h^{-1}	[0.099 0.099]	(Schulz, et al. 2017)	Human cell line
$\boldsymbol{k_{ph1}^{TRAF2}}$	TNF-α mediated phosphorylation rate of TRAF2	0.99 h^{-1}	fitted: (0.80 – 1.20) h^{-1}	[0.99 1.01]	-	
$\boldsymbol{k_{ph2}^{TRAF2}}$	CD40L mediated phosphorylation rate of TRAF2	k_{ph1}^{TRAF2}	-	-	-	
$\boldsymbol{k_{deg}^{TRAF2p}}$	degradation rate of phosphorylated TRAF2	1.03 h^{-1}	half-life 40 min: (0.832 – 1.248) h^{-1}	[1.03 1.05]	(Schulz, et al. 2017)	Human cell line
$\boldsymbol{k_{syn}^{IKK}}$	synthesis rate of IKK	k_{deg}^{IKK}	-	-	-	
$\boldsymbol{k_{deg}^{IKK}}$	Degradation rate of IKK	0.016 h^{-1}	fitted: (0.016 – 0.024) h^{-1}	[0.016 0.016]	-	
$\boldsymbol{k_{act}^{IKK}}$	activation rate of IKK mediated by phosphorylated IRAK1	67.19 h^{-1}	fitted: (67.19 – 100.8) h^{-1}	[67.19 67.19]	(Bode, Schroder, et al. 2007)	Mouse cell line
$\boldsymbol{k_{deg}^{IKKb}}$	degradation rate of active IKKβ	0.84 h^{-1}	fitted: (0.56 – 0.84) h^{-1}	[0.84 0.84]	-	
$\boldsymbol{k_{gain}^{NFkB}}$	gain rate of free NFκB in the nucleus mediated by IκBα and IKKβ	8.00 h^{-1}	fitted: (8 - 12) h^{-1}	[8.00 8.00]	-	

$k_{loss}^{NF\kappa B}$	the loss of free NF-κB mediated by IκBα	2.10 h^{-1}	half-life 2 h: fitted: (1.9 – 2.1) h^{-1}	[2.10 2.10]		
N_{tot}	total amount of free NF-κB	1	fixed	-	-	
k_{transc}^{mIkBa}	NF-κB mediated transcription rate of IκBα mRNA	1.52 h^{-1}	fitted: (1.52 – 2.28) h^{-1}	[1.52 1.52]	-	
k_{deg}^{mIkBa}	degradation rate of IκBαmRNA	1.05 h^{-1}	half-life 4 h: fitted: (0.95 – 1.05) h^{-1}	[1.05 1.05]	(Sinquett, et al. 2009)	Human cell line
k_{transl}^{IkBa}	translational rate of IκBα from IκBα mRNA	1.92 h^{-1}	fitted: (1.28 – 1.92) h^{-1}	[1.92 1.92]	-	
k_{loss}^{IkBa}	loss of free IκBα mediated by NF-κB and IKKβ	17.10 h^{-1}	half-life 30 min: fitted: (17.1 – 18.9) h^{-1}	[17.10 17.10]	(Sinquett, et al. 2009, Mathes, et al. 2008)	Human cell line
$k_{transc1}^{mIL12p35}$	basal transcription rate of IL-12p35 mRNA	fixed: 0	-	-	(Pfeiffer, et al. 2014)	Human cell line
$k_{transc2}^{mIL12p35}$	NF-κB mediated transcription rate of IL12p35 mRNA	0.012 h^{-1}	fitted: (0.01 ± 20%) h^{-1}	[0.012 0.012]	-	
$k_{deg}^{mIL12p35}$	degradation rate of IL12p35 mRNA	2.40 h^{-1}	fitted: (2 ± 20%) h^{-1}	[2.40 2.40]	-	
$k_{transl}^{IL12p70}$	translation rate of IL12p35 from its mRNA	$k_{deg}^{IL12p70}$	-	-	-	
$k_{deg}^{IL12p70}$	degradation rate of IL12p70	3.07 h^{-1}	fitted: (3 ± 20%) h^{-1}	[3.07 3.07]	-	
$k_{transc1}^{mIL6}$	basal transcription rate of IL-6 mRNA	fixed: 0	-	-	(Pfeiffer, et al. 2014)	Human cell line
$k_{transc2}^{mIL6}$	NF-κB mediated transcription rate of IL-6 mRNA	1.192 h^{-1}	fitted: (1 ± 20%) h^{-1}	[1.188 1.192]	-	
k_{deg}^{mIL6}	degradation rate of IL-6 mRNA	0.211 h^{-1}	half-life 4h: (0.2 ± 20%) h^{-1}	[0.210 0.211]	(Napolitani, et al. 2005)	Human cell line
k_{transl}^{IL6}	translation rate of IL-6 from its mRNA	k_{deg}^{IL6}	-	-	-	
k_{deg}^{IL6}	degradation rate of IL6	0.107 h^{-1}	half-life 16h: (0.09 ± 20%) h^{-1}	[0.107 0.107]	(Parola, et al. 2013)	Human cell line

$\boldsymbol{k_{transc1}^{mIL8}}$	basal transcription rate of IL-8 mRNA	fixed: 0	-	-	(Pfeiffer, et al. 2014)	Human cell line
$\boldsymbol{k_{transc2}^{mIL8}}$	NF-κB mediated transcription rate of IL-8 mRNA	0.558 h^{-1}	fitted: (0.5 ± 20%) h^{-1}	[0.541 0.558]	-	
$\boldsymbol{k_{deg}^{mIL8}}$	degradation rate of IL-8 mRNA	0.289 h^{-1}	half-life 2h: (0.35 ± 20%) h^{-1}	[0.289 0.289]	(Schulz, et al. 2017)	Human cell line
$\boldsymbol{k_{transl}^{IL8}}$	translation rate of IL-8 from its mRNA	k_{deg}^{IL8}	-	-		
$\boldsymbol{k_{deg}^{IL8}}$	degradation rate of IL8	0.162 h^{-1}	half-life 4h: (0.17 ± 10%) h^{-1}	[0.162 0.167]	(Schulz, et al. 2017)	Human cell line
$\boldsymbol{k_{transc1}^{mCD70}}$	basal transcription rate of CD70 mRNA	k_{deg}^{mCD70}	-	-	-	
$\boldsymbol{k_{transc2}^{mCD70}}$	NF-κB mediated transcription rate of CD70 mRNA	0.937 h^{-1}	fitted: (0.8 ± 20%) h^{-1}	[0.937 0.938]	-	Human cell line
$\boldsymbol{k_{deg}^{mCD70}}$	degradation rate of CD70 mRNA	0.0166 h^{-1}	fitted: (0.02 ± 20%) h^{-1}	[0.0166 0.0166]	-	Human cell line
$\boldsymbol{k_{transl}^{CD70}}$	translation rate of CD70 from its mRNA	k_{deg}^{CD70}	-	-	-	
$\boldsymbol{k_{deg}^{CD70}}$	Degradation rate of CD70	0.009 h^{-1}	fitted: (0.01 ± 20%) h^{-1}	[0.009 0.009]	-	Human cell line
$\boldsymbol{k_{biodist}^{IL12p70}}$	distribution rate of secreted IL-12p70 in blood	4 h^{-1}	half-life 10 min, fixed	-	(Kobayashi, et al. 2000)	Mouse experiment
$\boldsymbol{k_{sec}^{IL12p70}}$	secretion rate of IL12p70	$= k_{biodist}^{IL12p70}$	-	-	-	
$\boldsymbol{k_{biodist}^{IL6}}$	distribution rate of secreted IL-6 in blood	4 h^{-1}	half-life 10 min, fixed	-	(Kobayashi, et al. 2000)	Mouse experiment
$\boldsymbol{k_{sec}^{IL6}}$	secretion rate of IL-6	$k_{biodist}^{IL6}$	-	-	-	
$\boldsymbol{k_{biodist}^{IL8}}$	distribution rate of secreted IL-8 in blood	4 h^{-1}	half-life 10 min, fixed	-	(Kobayashi, et al. 2000)	Mouse experiment
$\boldsymbol{k_{sec}^{IL8}}$	secretion rate of IL-8	$k_{biodist}^{IL8}$	-	-	-	
$\boldsymbol{k_{deg,mod}^{IKKb}}$	degradation rate of IKKβ for caIKKβ-RNA electroporated DCs	0.216 h^{-1}	fitted: (0.18 ± 20%) h^{-1}	[0.216 0.216]		
$\boldsymbol{K_1, K_2, K_3}$	Michaelis-Menten like coefficients	fixed: 1	-	-	-	

Table S3. Model parameters for the DC-induced T-cell response.

Parameter	Description	Value	Range	95% CI	References	Notes
$\boldsymbol{k_{hom}^{N}}$	homing rate of naive T cells into spleen	k_{dist}^{N}	-	-	-	
$\boldsymbol{k_{dist}^{N}}$	distribution rate of naive cells from spleen into other compartments	0.029 h^{-1}	half-live ~ 24 h	-	(Henrickson, et al. 2008)	Mouse experiment
$\boldsymbol{k_{act}^{N}}$	activation rate of naive T cells	0.0087 h^{-1}	half-life ~ 8 h: 0.086 h^{-1}	-	(Nolz 2015, Ozga, et al. 2016)	Mouse experiment
$\boldsymbol{k_{prol}^{EE}}$	proliferation rate of early effector cells	0.029 h^{-1}	half-life ~ 24 h	-	(Crauste, et al. 2017)	Mouse experiment
$\boldsymbol{k_{diff1}^{EE}}$	differentiation rate of early effector T cells into short- lived effector cells	0.025 h^{-1}	half-life ~ 28 h	-	(Joshi, et al. 2007)	Mouse experiment
$\boldsymbol{k_{diff2}^{EE}}$	differentiation rate of early effector T cells into memory cells	0.025 h^{-1}	half-life ~ 28 h	-	(Joshi, et al. 2007)	Mouse experiment
$\boldsymbol{k_{deg}^{SLE}}$	degradation rate	$3.98 \cdot 10^{-4}$ h^{-1}	half-life (65 – 80) d: (3.6 - 4.4) $\cdot 10^{-4}$ h^{-1}	-	(Joshi, et al. 2007)	Mouse experiment
$\boldsymbol{N_0}$	initial value naive T cells in spleen	$1 \cdot 10^{6}$ cells	-	-	-	
$\boldsymbol{K_4}$	Michaelis-Menten like coefficient	577.5	fitted: 550 ± 5%	[577.5 577.5]	-	

Table S4. Sensitivity analysis results. The table shows first-order and total-order sensitivity indices of model parameters to the total amount of memory T cells and the corresponding confidence intervals of the sensitivity indices. The analyses were performed only on parameters that are relevant for T-cell response and parameters with fixed values during parameter estimation were not considered.

Parameter	First-order SI	Confidence Interval of first-order SI	Total-order SI	Confidence Interval of total-order SI
μ	0.032	[0.000 0.073]	0.012	[0.000 0.095]
μ_{BS}	0.034	[0.000 0.073]	0.020	[0.000 0.101]
μ_{S0}	0.019	[0.000 0.059]	0.000	[0.000 0.084]
DC_{in}	0.031	[0.000 0.070]	0.015	[0.000 0.097]
k_{deg}^{IRAK1}	0.019	[0.000 0.059]	0.000	[0.000 0.084]
k_{ph1}^{IRAK1}	0.019	[0.000 0.059]	0.000	[0.000 0.084]
k_{deg}^{IRAK1p}	0.019	[0.000 0.059]	0.000	[0.000 0.084]
k_{deg}^{TRAF2}	0.019	[0.000 0.059]	0.000	[0.000 0.084]
k_{ph1}^{TRAF2}	0.019	[0.000 0.059]	0.000	[0.000 0.084]
k_{deg}^{TRAF2p}	0.019	[0.000 0.059]	0.003	[0.000 0.087]
k_{deg}^{IKK}	0.081	[0.040 0.124]	0.085	[0.003 0.166]
k_{act}^{IKK}	0.019	[0.000 0.059]	0.000	[0.000 0.084]
k_{deg}^{IKKb}	0.216	[0.175 0.254]	0.212	[0.134 0.283]
k_{gain}^{NFkB}	0.030	[0.000 0.071]	0.008	[0.000 0.090]
k_{loss}^{NFkB}	0.023	[0.000 0.065]	0.003	[0.000 0.086]
k_{transc}^{mIkBa}	0.063	[0.021 0.104]	0.048	[0.000 0.129]
k_{deg}^{mIkBa}	0.091	[0.051 0.130]	0.085	[0.001 0.163]
$k^{I\kappa B\alpha}$	0.070	[0.029 0.112]	0.052	[0.000 0.134]
k_{loss}^{IkBa}	0.079	[0.039 0.120]	0.070	[0.000 0.149]
$k_{transc2}^{mIL12p35}$	0.027	[0.000 0.068]	0.012	[0.000 0.096]
$k_{deg}^{mIL12p35}$	0.019	[0.000 0.059]	0.000	[0.000 0.084]

$k_{deg}^{IL12p70}$	0.019	[0.000 0.060]	0.001	[0.000 0.085]
$k_{transc2}^{mIL6}$	0.034	[0.000 0.075]	0.018	[0.000 0.100]
k_{deg}^{mIL6}	0.036	[0.000 0.077]	0.022	[0.000 0.103]
k_{deg}^{IL6}	0.026	[0.000 0.067]	0.014	[0.000 0.097]
$k_{transc2}^{mIL8}$	0.034	[0.000 0.075]	0.013	[0.000 0.095]
k_{deg}^{mIL8}	0.039	[0.000 0.080]	0.027	[0.000 0.110]
k_{deg}^{IL8}	0.028	[0.000 0.069]	0.009	[0.000 0.094]
$k_{transc2}^{mCD70}$	0.019	[0.000 0.059]	0.000	[0.000 0.084]
k_{deg}^{mCD70}	0.019	[0.000 0.059]	0.000	[0.000 0.084]
k_{deg}^{CD70}	0.019	[0.000 0.059]	0.000	[0.000 0.084]
N_{tot}	0.156	[0.115 0.196]	0.160	[0.081 0.233]
k_{act}^{N}	0.110	[0.067 0.151]	0.123	[0.045 0.193]
k_{dist}^{N}	0.019	[0.000 0.059]	0.000	[0.000 0.083]
k_{prol}^{EE}	0.019	[0.000 0.059]	0.000	[0.000 0.084]
k_{diff1}^{EE}	0.019	[0.000 0.059]	0.000	[0.000 0.083]
k_{diff2}^{EE}	0.023	[0.000 0.064]	0.012	[0.000 0.094]
k_{deg}^{SLE}	0.019	[0.000 0.059]	0.000	[0.000 0.084]
K_4	0.032	[0.000 0.073]	0.012	[0.000 0.095]

Table S5. Parameters' first-order sensitivity indices for total amount of memory T cells over 200 h or 500 h simulation time. The last column indicates the changing of the ranking of parameters (down: less influential; up: more influential; -: unchanged).

Parameter	Sensitivity index 200 h	Sensitivity index 500 h	Ranking 200 h	Ranking 500 h	Remark
μ	0.032	0.024	14	19	Down
μ_{BS}	0.034	0.028	12	14	Down
DC_{in}	0.031	0.026	15	17	Down
k_{deg}^{IKK}	0.081	0.091	5	5	-
k_{deg}^{IKKb}	0.216	0.234	1	1	-
k_{transc}^{mIkBa}	0.063	0.077	8	7	Up
k_{deg}^{mIkBa}	0.091	0.100	4	4	-
$k^{I\kappa B\alpha}$	0.070	0.063	7	8	Down
k_{loss}^{IkBa}	0.079	0.080	6	6	-
$k_{transc2}^{mIL6}$	0.034	0.027	13	15	Down
k_{deg}^{mIL6}	0.036	0.032	10	11	Down
k_{deg}^{IL6}	0.026	0.029	19	12	Up
$k_{transc2}^{mIL8}$	0.034	0.036	11	9	Up
k_{deg}^{mIL8}	0.039	0.033	9	10	Down
N_{tot}	0.156	0.136	2	2	-
k_{act}^{N}	0.110	0.118	3	3	-
K_4	0.026	0.029	20	13	Up

Table S6. Parameters' first-order sensitivity indices for total amount of memory T cells when the estimated values of parameters are changed by 30% or 90%. The last column indicates the changing of the ranking of parameters (down: less influential; up: more influential; -: unchanged).

Parameter	Sensitivity index ± 30%	Sensitivity index ± 90%	Ranking ± 30%	Ranking ± 90%	Remark
μ	0.032	0.026	14	15	Down
μ_{BS}	0.034	0.022	12	19	Down
DC_{in}	0.031	0.028	15	11	Up
k_{deg}^{TRAF2p}	0.019	0.062	25	4	Up
k_{deg}^{IKK}	0.081	0.043	5	7	Down
k_{deg}^{IKKb}	0.216	0.132	1	2	Down
k_{transc}^{mIkBa}	0.063	0.031	8	10	Down
k_{deg}^{mIkBa}	0.091	0.039	4	8	Down
$k_{transl}^{I\kappa B\alpha}$	0.070	0.052	7	6	Up
k_{loss}^{IkBa}	0.079	0.032	6	9	Down
$k_{transc2}^{mIL6}$	0.034	0.022	13	18	Down
k_{deg}^{mIL6}	0.036	0.028	10	12	Down
$k_{transc2}^{mIL8}$	0.034	0.027	11	14	Down
k_{deg}^{mIL8}	0.039	0.025	9	16	Down
k_{deg}^{IL8}	0.028	0.027	17	13	Up
N_{tot}	0.156	0.075	2	3	Down
k_{act}^{N}	0.110	0.172	3	1	Up
k_{diff2}^{EE}	0.023	0.054	21	5	Up

3 References

Abbas, Abul K., Andrew H. Lichtman, and SHiv Pillai. 2018. *Cellular and molecular immunology.* 9. Elsevier.

Ahmed, Rafi, Michael J. Bevan, Steven L. Reiner, and Douglas T. Fearon. 2009. "The precursors of memory: models and controversies." *Nature Immunology* 9: 662-668. doi:10.1038/nri2619.

Arens, Ramon, Koen Schepers, Martijn A. Nolte, Michiel F. van Oosterwijk, René A. W. van Lier, Ton N. M. Schumacher, and Marinus H. J. van Oers. 2004. "Tumor Rejection Induced by CD70-mediated Quantitative and Qualitative Effects on Effector CD8+ T Cell Formation." *The Rockefelle University Press, J. Exp. Med.* 199: 1595-1605. doi:10.1084/jem.20031111.

Arsenio, Janilyn, Boyko Kakaradov, Patrick J. Metz, Stephanie H. Kim, Gene W. Yeo, and John T. Chang. 2013. "Early specification of CD8+ T lymphocyte fates during immunity revealed by single-cell gene-expression analyses." *Nature Immunology* 15: 365–372. doi:10.1038/ni.2842.

Badovinac, V. P., K. A. Messingham, A. Jabbari, J. S. Haring, and J. T. Harty. 2005. "Accelerated CD8+ T-cell memory and prime-boost response after dendritic-cell vaccination." *Nature Medicine* 11: 748–756. doi:10.1038/nm1257.

Badovinac, Vladimir P., Brandon B. Porter, and John T. Harty. 2002. "Programmed contraction of CD8+ T cells after infection." *Nature Immunology* (Springer Science and Business Media LLC) 3: 619–626. doi:10.1038/ni804.

Bode, Konrad A., Frank Schmitz, Leonardo Vargas, Klaus Heeg, and Alexander H. Dalpke. 2008. "Kinetic of RelA Activation Controls Magnitude of TLR-Mediated IL-12p40 Induction." *J. Immunology* 182: 2176–2184. doi:10.4049/jimmunol.0802560.

Bode, Konrad A., Kate Schroder, David A. Hume, Timothy Ravasi, Klaus Heeg, Matthew J. Sweet, and Alexander H. Dalpke. 2007. "Histone deacetylase inhibitors decrease Toll-like receptor-mediated activation of proinflammatory gene expression by impairing transcription factor recruitment." *Immunology* 122: 596–606. doi:10.1111/j.1365-2567.2007.02678.x.

Borst, Jannie, Jenny Hendriks, and Yanling Xiao. 2005. "CD27 and CD70 in T cell and B cell activation." *Elsevier, Current Opinion in Immunology* 17: 275–281. doi:10.1016/j.coi.2005.04.004.

Celli, Susanna, Mark Day, Andreas J. Müller, Carmen Molina-Paris, Grant Lythe, and Philippe Bousso. 2012. "How many dendritic cells are required to initiate a T-cell response." *Blood* 120: 3945–3948. doi:10.1182/blood-2012-01-408260.

Crauste, Fabien, Julien Mafille, Lilia Boucinha, Sophia Djebali, Olivier Gandrillon, Jacqueline Marvel, and Christophe Arpin. 2017. "Identification of Nascent Memory CD8 T Cells and Modeling of Their Ontogeny." *Cell Systems* 4: 306–317. doi:10.1016/j.cels.2017.01.014.

Girel, Simon, Christophe Arpin, Jacqueline Marvel, Olivier Gandrillon, and Fabien Crauste. 2007. "Model-Based Assessment of the Role of Uneven Partitioning of Molecular Content on Heterogeneity and Regulation of Differentiation in CD8 T-Cell Immune Responses." *Frontiers in Immunology* 10. doi:10.3389/fimmu.2019.00230.

Henrickson, Sarah E., Thorsten R. Mempel, Irina B. Mazo, Bai Liu, Maxim N. Artyomov, Huan Zheng, Antonio Peixoto, et al. 2008. "T cell sensing of antigen dose governs interactive behavior with dendritic cells and sets a threshold for T cell activation." *Nat Immunol.* 9: 282–291. doi:10.1038/ni1559.

Henry, Curtis J., David A. Ornelles, Latoya M. Mitchell, Kristina L. Brzoza-Lewis, and Elizabeth M. Hiltbold. 2008. "IL-12 Produced by Dendritic Cells Augments CD8+ T cell Activation through the Production of the Chemokines CCL1 and CCL17." *J. Immunol.* 181: 8576–8584. doi:10.4049/jimmunol.181.12.8576.

Hunter, Christopher A., and Simon A. Jones. 2017. "IL-6 as a keystone cytokine in health and disease." *Nature Immunology* 16: 448-457. doi:10.1038/ni.3153.

Jenkins, Marc K., H. Hamlet Chu, James B. McLachlan, and James J. Moon. 2010. "On the composition of the preimmune repertoire of T cells specific for peptide-major histocompatibility complex ligands." *Annu. Rev. Immunol.* 28: 275–294. doi:10.1146/annurev-immunol-030409-101253.

Joshi, Nikhil S., Weiguo Cui, Anmol Chandele, Heung Kyu Lee, David R. Urso, James Hagman, Laurent Gapin, and Susan M. Kaech. 2007. "Inflammation Directs Memory Precursor and Short-Lived Effector CD8(+) T Cell Fates via the Graded Expression of T-bet Transcription Factor." *Immunity* 27: 281–295. doi:10.1016/j.immuni.2007.07.010.

Kobayashi, H., J. A. Carrasquillo, C. H. Paik, T. A. Waldmann, and Y. Tagaya. 2000. "Differences of biodistribution, pharmacokinetics, and tumor targeting between interleukins 2 and 15." *Cancer research* 60 (13): 3577–3583.

Ludewig, Burkhard, Philippe Krebs, Tobias Junt, Helen Metters, Neville J. Ford, Roy M. Anderson, and Gennady Bocharov. 2004. "Determining control parameters for dendritic cell-cytotoxic T lymphocyte interaction." *Wiley-VCH Verlag GmbH and Co. KGaA, Weinheim, European Journal of Immunology* 34: 2407–2418. doi:10.1002/eji.200425085.

Mackensen, Andreas, Thomas Krause, Uli Blum, Peter Uhrmeister, Roland Mertelsmann, and Albrecht Lindemann. 1999. "Homing of intravenously and intralymphatically injected human dendritic cells generated in vitro from CD34+ hematopoietic progenitor cells." *Springer Verlag, Cancer Immunology, Immunotherapy* 48: 118–122. doi:10.1007/s002620050555.

Mathes, Erika, Ellen L. O'Dea, Alexander Hoffmann, and Gourisankar Ghosh. 2008. "NF-kappaB dictates the degradation pathway of IkappaBalpha." *EMBO J* 27: 1357–67. doi:10.1038/emboj.2008.73.

Mueller, S. N., T. Gebhardt, F. R. Carbone, and W. R. Heath. 2013. "Memory T cell subsets, migration patterns, and tissue residence." *Annu. Rev. Immunol.* 31: 137–161. doi:10.1146/annurev-immunol-032712-095954.

Napolitani, Giorgio, Andrea Rinaldi, Francesco Bertoni, Federica Sallusto, and Antonio Lanzavecchia. 2005. "Selected Toll-like receptor agonist combinations synergistically trigger

a T helper type 1-polarizing program in dendritic cells." *Nat Immunol.* 6: 769–76. doi:10.1038/ni1223.

Nolz, Jeffrey C. 2015. "Molecular mechanisms of CD8+ T cell trafficking and localization." *Cell Mol Life Sci* 72: 2461–2473. doi:10.1007/s00018-015-1835-0.

Ozga, Aleksandra J., Federica Moalli, Jun Abe, Jim Swoger, James Sharpe, Dietmar Zehn, Mario Kreutzfeldt, Doron Merkler, Jorge Ripoll, and Jens V. Stein. 2016. "pMHC affinity controls duration of CD8+ T cell–DC interactions and imprints timing of effector differentiation versus expansion." *J. Exp. Med.* 219: 2811–2829. doi:10.1084/jem.20160206.

Parker, Belinda S., Jai Rautela, and Paul J. Hertzog. 2016. "Antitumour actions of interferons: implications for cancer therapy." *Nature Reviews, Cancer* 16: 131-144. doi:10.1038/nrc.2016.14.

Parola, Carmen, Laura Salogni, Xenia Vaira, Sara Scutera, Paolo Somma, Valentina Salvi, Tiziana Musso, et al. 2013. "Selective activation of human dendritic cells by OM-85 through a NF-kB and MAPK dependent pathway." *PLoS One* 8. doi:10.1371/journal.pone.0082867.

Pearce, Erika L., and Hao Shen. 2007. "Generation of CD8 T Cell Memory Is Regulated by IL-12." *J. Immunol.* 179: 2074–2081. doi:10.4049/jimmunol.179.4.2074.

Pfeiffer, Isabell A., Stefanie Hoyer, Kerstin F. Gerer, Reinhard E. Voll, Ilka Knippertz, Eva Gückel, Gerold Schuler, Niels Schaft, and Jan Dörrie. 2014. "Triggering of NF-κB in cytokine-matured human DCs generates superior DCs for T-cell priming in cancer immunotherapy." *European Journal of Immunology* 44: 3413–3428. doi:10.1002/eji.201344417.

Pianosi, Francesca, Fanny Sarrazin, and Thorsten Wagener. 2015. "A Matlab toolbox for Global Sensitivity Analysis." *Environmental Modelling & Software* 70: 80-85. doi:10.1016/j.envsoft.2015.04.009.

Pianosi, Francesca, Keith Beven, Jim Freer, Jim W. Hall, Jonathan Rougier, David B. Stephenson, and Thorsten Wagener. 2016. "Sensitivity analysis of environmental models: A systematic review with practical workflow." *Environmental Modelling & Software* 79: 214–232. doi:10.1016/j.envsoft.2016.02.008.

Saltelli, A., M. Ratto, T. Andres, F. Campolongo, J. Cariboni, D. Gatelli, M. Saisana, and S. Tarantola. 2008. *Global Sensitivity Analysis. The Primer.* 3. Wiley Interscience.

Sarrazin, Fanny, Francesca Pianosi, and Thorsten Wagener. 2016. "Global Sensitivity Analysis of environmental models: Convergence and validation." *Environmental Modelling & Software* 79: 135–152. doi:10.1016/j.envsoft.2016.02.005.

Sauermost, Rolf, and Doris Freudig. 1999. "Blutspeicher." *Lexikon der Biology, Spektrum.* https://www.spektrum.de/lexikon/biologie/blutspeicher/9696.

Schulz, Christine, Xin Lai, Wilhelm Bertrams, Anna Lena Jung, Alexandra Sittka-Stark, Christina Elena Herkt, Harshavadhan Janga, et al. 2017. "THP-1-derived macrophages render lung epithelial cells hypo-responsive to Legionella pneumophila - a systems biology study." *Scientific Reports* 7. doi:10.1038/s41598-017-12154-4.

Sinquett, Frank L., Rebecca L. Dryer, Valentina Marcelli, Ameesha Batheja, and Lori R. Covey. 2009. "Single nucleotide changes in the human Igamma1 and Igamma4 promoters underlie different transcriptional responses to CD40." *J. Immunology* 182: 2185-2193. doi:10.4049/jimmunol.0802700.

Tannenbaum, Charles S., and Thomas A. Hamilton. 2000. "Immune-inflammatory mechanisms in IFNγ -mediated anti-tumor activity." *Cancer Biology* 10: 113–123. doi:10.1006/scbi.2000.0314.

Taub, Dennis D., Miriam Anver, Joost J. Oppenheim, Dan L. Longo, and William J. Murphy. 1996. "T Lymphocyte Recruitment by Interleukin-8 (IL-8)." *The Journal of Clinical Investigation* 97: 1931–1941. doi:10.1172/JCI118625.

Vecchio, Michele Del, Emilio Bajetta, Stefania Canova, Michael T. Lotze, Amy Wesa, Girogio Parmiani, and Andrea Anichini. 2007. "Interleukin-12: Biological properties and clinical application." *Clin Cancer Res* 13: 4677-4685. doi:10.1158/1078-0432.CCR-07-0776.

Vrisekoop, N., den Braber I., de Boer A.B., A. F. Ruiter, M. T. Ackermans, van der Crabben S.N., E. H. Schrijver, et al. 2008. "Sparse production but preferential incorporation of recently produced naive T cells in the human peripheral pool." *Proc. Natl. Acad. Sci. USA* 105: 6115–6120. doi:10.1073/pnas.0709713105.

Wherry, E. John, Volker Teichgraeber, Todd C. Becker, David Masopust, Susan M. Kaech, Rustom Antia, Ulrich H. von Andrian, and Rafi Ahmed. 2003. "Lineage relationship and protective immunity of memory CD8 T cell subsets." *Nature Immunology* 4: 225–234. doi:10.1038/ni889.
